# To Coke or Not to Coke: When Pd Is Not Noble Anymore under Methane Dry Reforming Conditions

**DOI:** 10.1021/acscatal.5c07296

**Published:** 2025-12-08

**Authors:** Mahdi Hosseinpour, Thomas F. Winterstein, Clivia Hejny, Marc Heggen, Bernhard Klötzer, Simon Penner

**Affiliations:** † Institute of Physical Chemistry, 27255 University of Innsbruck, Innrain 52c, 6020 Innsbruck, Austria; ‡ Institute of Mineralogy and Petrography, University of Innsbruck, Innrain 52f, 6020 Innsbruck, Austria; § Ernst Ruska-Centre for Microscopy and Spectroscopy with Electrons, 28334 Forschungszentrum Jülich GmbH, Leo-Brandt-Str. 1, D-52428 Jülich, Germany

**Keywords:** dry reforming of methane, palladium−zirconium catalysts, catalyst regeneration, metal−oxide phase boundary, carbon deposition, tip-growth mechanism

## Abstract

We explore the fundamental pathways of carbon formation and regeneration in a model Pd/Zr catalyst during dry reforming of methane (DRM) and under related reaction conditions. Using a combination of XPS, SEM, and EDX, we track the structural and chemical changes of the catalyst throughout the reaction, deactivation, and regeneration cycles. By systematically adjusting feed composition, CO_2_ conversion, and regeneration atmospheres, the study identifies how different gas-phase species contribute to carbon formation and clean-off. It also determines the conditions that influence the accessibility of the reactive metal–oxide interfaces. A comparison with the analogous Ni/Zr system highlights how the choice of the metal affects regeneration chemistry and the importance of accessible metal oxide phase boundaries in CO_2_ activation. The experimental setup combines temperature-resolved reaction profiling with micro- and spectroscopic surface characterization at key intermediate stages, enabling direct links among catalytic activity, surface morphology, and regeneration results. This approach offers insights into how catalyst design, operational conditions, and regeneration methods can be optimized to achieve high DRM activity and effective carbon management in noble metal–oxide systems.

## Graphical abstract

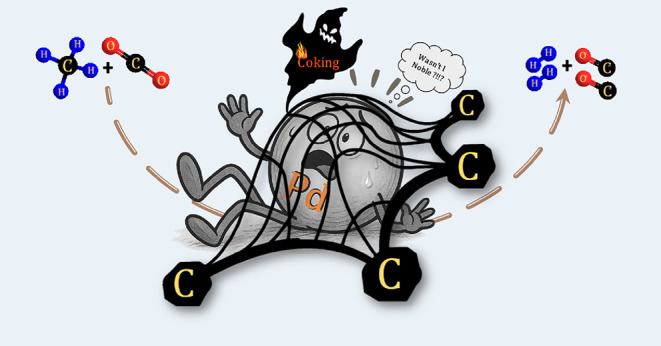

## 1 Introduction

Dry reforming of methane (DRM), CH_4_ +  CO_2_ → 2H_2_ + 2CO, is an attractive catalytic process for simultaneously converting two strong greenhouse gases into synthesis gas (syngas), a feedstock for Fischer–Tropsch and other chemical syntheses.^1–4^ With a theoretical H_2_/CO ratio of unity, DRM is particularly relevant for producing syngas customized for oxygenate and hydrocarbon synthesis.^5–7^ However, despite its promise, DRM continues to face three major obstacles for industrial arrangement: strong catalyst deactivation due to carbon deposition (coking), sintering of active metal particles under high-temperature operation, and the difficulty of balancing high conversion with long-term stability. Work by Zhong and colleagues highlights the difficulty of achieving high conversion while maintaining long-term catalyst stability, noting that conditions optimizing conversion often accelerate coking or sintering.^8^ Carbon deposition remains the most critical obstacle, as it rapidly deactivates catalysts, especially Ni-based systems, through extensive coke buildup, overshadowing other limitations in DRM processes.^9^ For example, a study by Wei et al. emphasizes that carbon deposition (coking) and metal sintering are widely acknowledged as the primary causes of catalyst deactivation in Ni-based DRM systems. At typical operating temperatures (500–900 °C), carbon whiskers and graphitic carbon form and envelop active sites, while sintering reduces metal dispersion and active surface area.^10^ Methane decomposition is often identified as the primary source of coke. However, the reaction environment itself adds aspects of complexity, as rapid methane dehydrogenation can accelerate the buildup of surface carbon, and the equilibrium between carbon formation and oxidation under CO_2_-rich conditions critically influences catalyst stability.^9,11,12^ CO disproportionation (2 CO­(g) → C(s) + CO_2_(g)) and even secondary reactions involving the product gases (CO­(g) + H_2_(g) → C(s) + H_2_O­(g)) can also lead to carbon formation, especially in reaction environments where high concentrations of the products remain in extended contact with the catalyst surface.^1^ These overlapping pathways create a narrow operational window where activity and stability must be carefully balanced to avoid rapid deactivation, as optimal DRM performance emerges only under tightly controlled temperature, space velocity, and feed ratios. Outside this window, side reactions lead to coke or sintering, selectivity loss with respect to H_2_,^13^ and quick catalyst failure.^14,15^ Both noble and base metals have been explored as active components for DRM, with base metals such as Fe, Co, and Ni offering high activity at low cost, and noble metals like Rh, Pt, Ru, and Pd delivering superior stability and anticoking performance, often influencing bimetallic designs to combine the advantages of both classes.^16,17^ Nickel-based catalysts, despite their cost advantage and high activity for CH_4_ activation, are notably prone to severe coking and sintering, requiring strategies such as the use of high-surface-area supports, promoters, or engineered metal–oxide interfaces.^18–21^ Noble metals, particularly palladium (Pd), have been investigated as an alternative due to their unique electronic structure, lower tendency toward bulk carbide formation, and potential to moderate carbon–metal interactions^22^ (e.g., a mechanochemical Pd/CeO_2_ catalyst shows enhanced DRM activity and reduced coking or reviews emphasizing Pd’s lower carbide formation and moderated carbon–metal bonding^23^). Pd-based catalysts are empirically described in the literature as more resistant to coking compared to Ni-based systems,^24,25^ although the mechanistic basis of this insight and its dependence on reaction conditions remain insufficiently explored from the experimental side, given that DFT studies report weaker carbon adsorption on Pd(111) versus Ni(111)^26^

Support selection plays a critical role in shaping both activity and coking behavior, as demonstrated in comparisons of high-surface-area supports (γ-Al_2_O_3_,^27^ modified spinels,^27^ praseodymia-ceria,^28^ zeolites^19^) that promote stronger metal–support interactions, provide oxygen mobility for gasifying carbon deposits, and support sustained DRM activity with reduced coke formation over extended operation.^29^ Zirconia (ZrO_2_), with its thermal stability and redox properties, has emerged as a promising support for both Ni and Pd systems.^30–33^ Next to activation, these materials undergo surface restructuring, forming Pd/ZrO_2_ interfaces, whose electronic and structural characteristics can influence CH_4_ activation and CO_2_ dissociation, as evidenced by in situ NAP-XPS and XRD studies of our group showing carbide-modified Pd^0^ nanoparticles with active interfaces to ZrO_2_ (enhancing CO_2_ activation).^34,35^ Complementary first-principles modeling indicates oxygen-vacancy-mediated interface effects on methane and carbon dioxide activation.^36,37^ Importantly, such metal oxide phase boundaries have been proposed as key sites for CO_2_-assisted carbon removal via the reverse Boudouard reaction, especially in Ni-based systems, since well-dispersed Ni near ZrO_2_ facilitates carbon spillover and efficient gasification of deposited carbon by CO_2_, helping to sustain activity during cyclic dry reforming conditions.^22^ Whether Pd-based catalysts can utilize similar interfacial chemistry or whether their response to DRM and regeneration environments is fundamentally different is an open question that carries both mechanistic and practical significance.

In this work, we use a well-defined intermetallic Pd/Zr precatalyst model system to uncover the fundamental pathways of carbon formation and removal during DRM. Intermetallic-based precursors offer the additional advantage of high electronic conductivity, which makes them ideally suited for electron spectroscopic and microscopic methodologies (e.g., NAP-XPS, SEM/EDX). This allows us to directly track structural and chemical dynamics under relevant reaction conditions, an aspect that is often inaccessible with conventional oxide-based catalysts. By combining XPS, SEM/EDX, and controlled batch reactor experiments, we track the structural and chemical evolution of the catalysts across multiple DRM cycles and regeneration treatments. We investigate the gas-phase origin of coke under high conversion conditions, examining the role of syngas products in carbon formation, and evaluate the effectiveness of both CO_2_- and O_2_-based regeneration protocols. Through direct comparison with the initially intermetallic Ni/Zr system, we clarify how the presence or absence of active metal oxide phase boundaries governs regenerative behavior and ultimately propose a mechanistic framework for designing Pd-based DRM catalysts capable of achieving optimum conversion with controlled carbon management.

## 2 Materials and Methods

### 2.1 Catalyst Preparation

The intermetallic Pd–Zr sample was prepared by physical vapor deposition (PVD), utilizing small pieces of pure palladium wire (high purity, 1 mm thickness) and zirconium foil (Alpha Aesar, 99.5% purity, 0.1 mm thickness) under high-vacuum conditions (1·10^–6^ mbar). Before deposition, the Zr foil was cleaned mechanically to remove oxide layers while being immersed in a heptane bath to avoid surface reoxidation by air contact. The deposition of thin Pd films on Zr was carried out via thermal evaporation by using a highly adaptable modular high-vacuum chamber. The chamber is based on a Schott Duran flat flange system with glass recipients.^38^ The setup includes home-built stainless steel ring modules compatible with standard glass flat flanges from Schott DN100 (inner diameter: 90 mm). Two metal rings, separated by fluorocarbon O-rings (diameter: 99 mm) and sealed with high-vacuum grease, were used instead of multiple inspection windows to maintain simplicity, utilizing a glass recipient. A quartz crystal microbalance with QS 008 Ag quartz crystals (Umicore) monitors the coating thickness of the thin film at an ∼5 MHz resonance frequency. A home-built substrate mount holds the Zr foil substrate (18 × 20 mm^2^). The tungsten boat for thermal-resistive heating is positioned below the substrate mount. A PKR 361 Pirani/cold cathode gauge (Pfeiffer Vacuum AG, Aßlar, Germany) is used for pressure monitoring. The chamber is pumped by a turbomolecular pump (300 L s^–1^) and a two-stage rotary vane vacuum pump, maintaining a base pressure in the low 10^–7^ mbar regime. Electrical feedthroughs connect the substrate mount and the boat suspension to a low-voltage/high-current power supply. During deposition, the substrate mount was heated to 170 °C, enhancing the adhesion and growth properties of the film. The entire setup, including the quartz crystal microbalance, boat suspension, and transformer connectors, is water-cooled. Before the DRM experiments, the Pd-coated surface of the Zr foil was transformed into a Pd–Zr bimetallic alloy layer within the UHV XPS/LEIS/AES chamber, as detailed in Section 2.2. The cleaning process and subsequent alloy formation involved Ar+ sputtering (5 × 10^–5^ mbar Ar, 2 keV, 1 μA sample current) and gradual thermal annealing from 25 to 800 °C under UHV conditions (5 × 10^–10^ mbar), respectively. This treatment continued until XPS spectra indicated a saturated final alloy surface composition of ∼50 atom % Pd and ∼50 atom % Zr as the target catalyst composition for DRM application (Figure 1). An ultraclean Pd foil (Goodfellow, 99.95% purity, 0.1 mm thickness, 18 × 20 mm^2^) was used as a reference catalyst. The surface cleaning process for the Pd foil followed the same steps as for the Pd–Zr foil.

**Figure 1:**
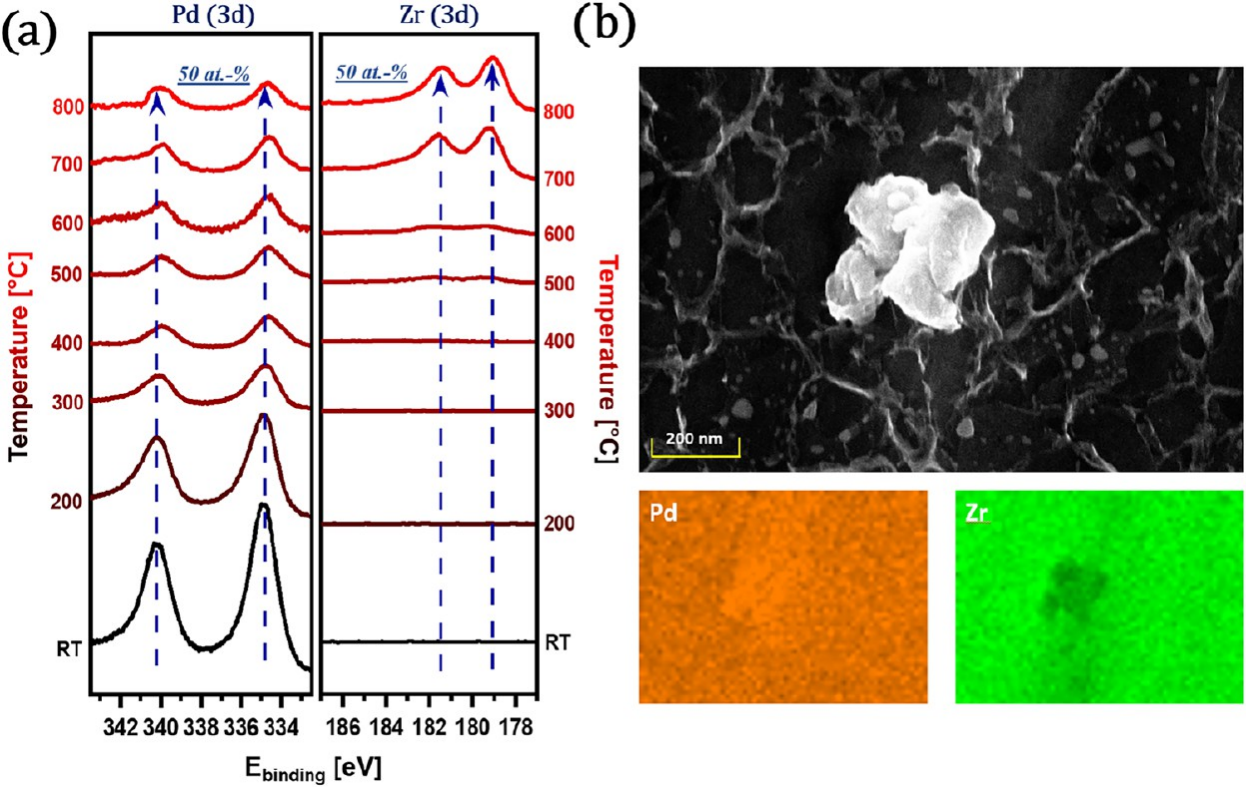
(a) Surface chemical evolution of the initial 100 nm Pd-on-Zr layer during step-by-step annealing from 25 to 800 °C, monitored by XPS. (b) SEM image and EDX elemental maps of the Pd–Zr alloy surface after annealing to 800 °C, before DRM testing.

### 2.2 UHV System with Attached High-Pressure Batch Reaction Cell

The UHV system, equipped with an all-quartz recirculating batch reactor, is thoroughly detailed in ref 39 and engineered for quantitative catalytic and kinetic studies up to 1 bar on polycrystalline foils, with product detection either by continuous online MS analysis (HP GC-MS System G1800A) through a capillary leak, or by intermittent column injection GC-MS analysis. MS signals of CH_4_, CO_2_, and CO (*m*/*z* 15 + 16, 44, and 28) were externally calibrated and corrected for fragmentation. All DRM reactions were performed with initial partial pressures of 50 mbar of CH_4_ and 50 mbar of CO_2_. The reaction cell was backfilled with pure He to a total pressure of 1013 mbar to ensure efficient gas intermixing through recirculation and to enhance heat transfer to the sample by improving thermal conductivity. For the DRM tests, the reactor was heated at a constant rate of 10 °C min^–1^ to a final temperature of 800 °C, with an isothermal section for 30 min.

For quantification of the catalytic profiles, we have accordingly calculated the turnover number (TON) and turnover frequency (TOF) for the pure Pd sample as a reference and for the Pd/Zr catalyst based on CO TPD results and batch reactor data. To determine the number of Pd sites, we performed CO adsorption at 25 °C and subsequent quantitative temperature-programmed desorption (TPD) on both samples. Detailed calculations and explanations, along with the TPD profiles, are provided in the Supporting Information (Figure S1 and Equation S1). The average TOF value of ∼8 s^–1^ per Pd surface atom indicates highly efficient catalyst turnover under batch reactor conditions.^40^ Also, maximum TOF values were extracted from the individual inflection points of the TON vs time curves’ first derivatives and summarized in Table 1 of the SI.

### 2.3 Scanning Electron Microscopy (SEM)

Scanning electron microscopy and corresponding energy-dispersive X-ray spectroscopy (EDXS) measurements were performed on a field-free analytical TESCAN Clara ultrahigh-resolution scanning electron microscope operated at 10 kV with a beam current of 1.0 nA. EDX maps were collected using an Oxford Ultim Max 65 mm^2^ detector. Further SEM and EDX analysis was conducted on a Zeiss GeminiSEM 460 equipped with an Oxford Ultim Max 170 mm^2^ analytical silicon drift detector operated at 20 kV.

### 2.4 X-ray Photoelectron Spectroscopy (XPS)

All XP spectra were collected using a Thermo Fisher α 110 hemispherical analyzer and SPECS XR 50 twin Mg/Al Kα X-ray gun attached to the UHV chamber. Mg Kα radiation was used in all experiments. All XPS data were analyzed using the CasaXPS software program, version 2.3.25 PR1.0. For peak fitting, a Shirley background was applied to all spectra. Deconvolution of all Zr 3d spectra included the metallic Zr^0^ component at a binding energy (BE) of 179.1 eV and ZrO_2_ at a BE of 183.0 eV.^41,42^ The Pd 3d_5/2_ peak is assigned to a binding energy (BE) of 335.0 eV for the metallic component and PdO at a BE of 336.4 eV.^34^ The C 1s BE values are 284.4 eV for graphitic carbon and approximately 282.2 eV for carbide-type carbon.^43^ To fit the O 1s spectrum, three different components were considered: ZrO_2_ at 530.2 eV, Zr suboxide at 531.2 eV, and hydroxylated zirconium species at 531.4 eV.^44^

### 2.5 Error Analysis and Statistical Treatment

All quantitative values reported in this work are presented as mean ± standard deviation (SD). Particle size distributions were extracted from at least *n* = 100–150 particles per image using the program ImageJ. Atomic surface compositions (XPS) represent the mean of at least three independent measurements taken at different positions on the sample and include an estimated XPS quantification error of ±2–3 at. %. CO_2_ conversion values were derived from repeated DRM cycles (*n* = 4), and the variation across cycles is reported as SD. These statistical descriptors provide an estimate of measurement reproducibility and natural variation within the model catalyst system.

## 3 Results and Discussion

### 3.1 Establishing a Model Material to Distinguish Different Routes of Coke Formation

Figure 1a shows the evolution of Pd 3d and Zr 3d XP spectra during stepwise annealing of the as-prepared Pd/Zr system (from 25 °C to 800 °C). At room temperature, the surface is dominated by Pd, as reflected by the strong Pd 3d signal. With increasing temperature, this signal gradually diminishes, indicating Pd diffusion into the underlying Zr substrate. Simultaneously, the Zr 3d signal grows, reflecting the enrichment of Zr at the surface due to metal interdiffusion. Above ∼700 °C, the Pd and Zr signals stabilize, reaching an approximate surface composition of 50 at.% Pd and 50 at.% Zr at 800 °C.

These observations demonstrate the formation of a Pd–Zr intermetallic phase via thermally driven alloying in ultrahigh vacuum. This process occurs even in the absence of reactive gases, emphasizing the intrinsic tendency of Pd–Zr systems to undergo significant surface restructuring at elevated temperatures. Such transformations are critical to consider when probing coke formation pathways under the reaction conditions. For clarity, the as-deposited Pd/Zr surface before annealing is shown in Figure S2, confirming the dense, granular Pd overlayer and uniform elemental distribution before thermal intermixing.

Figure 1b provides complementary SEM and EDX elemental mapping of the Pd/Zr surface after annealing to 800 °C under vacuum conditions (*p* ∼ 5 × 10^–9^ mbar) and exposure to air (for SEM imaging). The SEM image and the EDX maps reveal mostly a continuous, textured surface morphology and elemental distribution characteristic of an intermetallic phase with some discrete Pd islands and ZrO_2_ arising from air corrosion of the top layers of Pd–Zr. Note that the state presented in Panel (b) does not directly correlate with the XP spectrum at 800 °C shown in Panel (a), as under UHV conditions, the corrosion does not take place, and accordingly, the Zr 3d spectra do not show an oxide component. This observed morphology and composition suggest that high-temperature annealing drives not only atomic interdiffusion but also the macroscale reorganization of the surface. Such a homogeneous Pd–Zr intermetallic state serves as an ideal model system for subsequent reactivity studies.

### 3.2 Structure–Activity—Coking Correlation during DRM

#### 3.2.1 Building a Clean Pd/Zr Model: Investigation of Carbon Formation by CH_4_ Decomposition

Exposure of the UHV-annealed sample to pure CH_4_ leads to a distinct change in morphology and the formation of surface Pd nanoparticles acting as active sites for CH_4_ decomposition and carbon growth. (Figure 2). Monitoring the methane pressure during heating (Figure 2a) reveals a steady consumption above 700 °C, with a significant slowdown during the isothermal period at 800 °C. This behavior is consistent with methane decomposition, resulting in the deposition of carbon on the catalyst surface. After reaction, the sample showed visible darkening (“carbon black”), confirming macroscopic carbon accumulation, which is further supported by SEM evidence of filamentous and amorphous carbon structures. Postreaction SEM imaging (Figure 2b) shows a dense network of carbon filaments with Pd nanoparticles located at the tips, consistent with the classical tip-growth mechanism of filamentous carbon. In this process, CH_4_ decomposition supplies carbon that dissolves into the Pd particle, diffuses through it, and subsequently precipitates at the interface between Pd and the support, driving filament elongation and leaving the Pd particle positioned at the tip. The high-resolution inset displays tip-located Pd particles in more detail, highlighting their active role in catalyzing filamentous carbon growth through a local, bulk diffusion-induced concentration gradient. EDX mapping confirms this observation, showing the complementary Pd and C signals, whereby the Pd particles appear carbon-depleted, while Zr remains largely buried.

**Figure 2:**
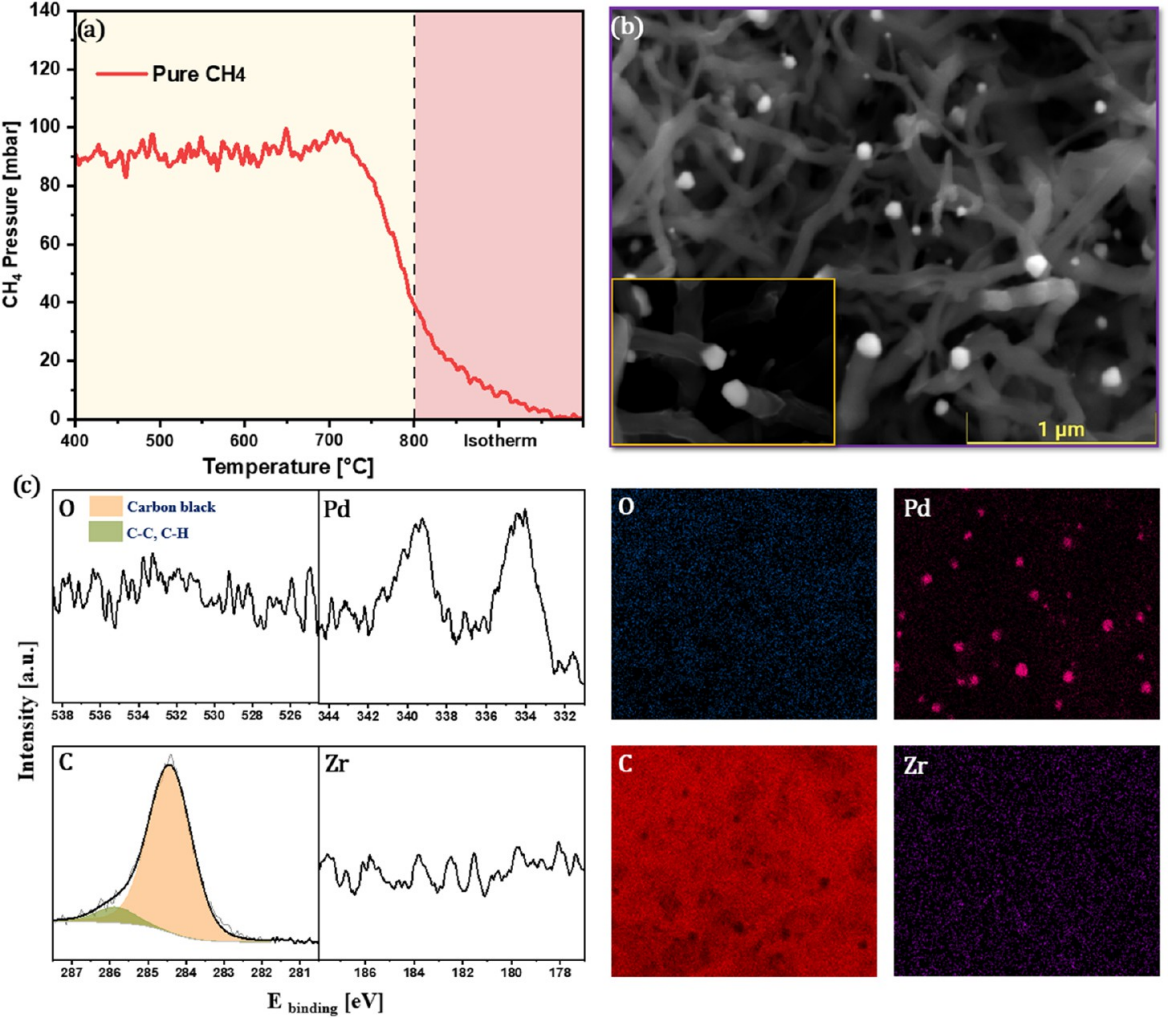
(a) Temperature-programmed decomposition profile of pure CH_4_ on the Pd/Zr catalyst. (b) SEM image and corresponding EDX elemental maps after CH_4_ decomposition. (c) XPS survey spectra of the surface after CH_4_ decomposition. Reaction conditions: 100 mbar CH_4_, linear temperature ramp (10 °C min^–1^) up to 800 °C, followed by isothermal reaction for 30 min.

XPS analysis (Figure 2c) further supports these findings, with strong carbon signals dominating the spectra and significantly weakened O 1s and Zr 3d signals. This suggests a massive carbon overlayer suppressing photoelectron emission from the underlying alloy. These observations suggest that Pd–Zr surfaces readily catalyze methane cracking at elevated temperatures, resulting in extensive coke formation.

While CH_4_ decomposition highlights the intrinsic tendency of surface-activated Pd/Zr to promote carbon buildup, this occurs because CH_4_ dissociation on metallic Pd generates surface carbon atoms, together with hydrogen. This simple decomposition environment does not reflect the more complex environment of dry reforming of methane (DRM), where CH_4_ and CO_2_ are cofed. Introducing CO_2_ into the system can suppress coking via in situ gasification of carbon or create entirely new surface dynamics due to the presence of oxygenated intermediates/products. To explore these effects, we investigated the UHV-annealed Pd/Zr catalyst under stoichiometric DRM conditions (CH_4_:CO_2_ = 1:1).

#### 3.2.2 Structural Evolution of Pd/Zr during DRM and Onset of Carbon Formation

The evolution of the initially intermetallic Pd/Zr catalyst under dry reforming conditions (CH_4_:CO_2_ = 1:1) was followed in a stepwise fashion, correlating the catalytic activity with structural and compositional changes. Figure 3a shows the CO_2_ conversion profile during the first DRM cycle, with the conversion rising sharply above 500 °C and approaching nearly 100 ± 1% at 800 °C. To capture structural transformations during this activation period, SEM and EDX analyses were performed at four crucial temperatures (350, 500, 650, and 800 °C). At 350 °C (Figure 3a.1), rather than discrete Pd particles, this observation may indicate the early formation of a thin ZrO_2_-rich overlayer, resulting from increasing Pd–Zr decomposition that partially shields Pd from detection. Such behavior could arise from the onset of surface reconstruction, potentially involving Pd–Zr interdiffusion or ZrO_2_ segregation. This interpretation aligns with the homogeneous EDX contrast, suggesting that Pd remains largely buried beneath the growing ZrO_2_-rich surface layer at this stage. By 500 °C (Figure 3a.2), the surface begins to fracture, revealing discrete Pd particles emerging through cracks within the ZrO_2_-rich overlayer. EDX mapping further confirms the identity of these protrusions as Pd, highlighting their localization within the reconstructed surface. At 650 °C (Figure 3a.3), the catalyst reaches a critical point, entering the so-called “coking window”. Early indications of carbon deposition emerge, visible as filamentous structures sprouting from Pd particles. Yellow arrows in the SEM image highlight the initial carbon filaments. Complementary EDX carbon mapping supports this observation, confirming the initial stages of coke formation on the Pd/Zr surface even before the system reaches the isothermal DRM conditions. Finally, at 800 °C (Figure 3a.4), the surface is dominated by dense networks of carbon filaments connected to Pd particles. This morphological shift reflects the dual nature of high catalytic activity: although almost complete CO_2_ conversion is achieved, it comes at the cost of significant coking, leading to catalyst deactivation risks. Figure 3b complements these findings with quantitative XPS analysis. The atomic percentages reveal a progressive surface enrichment of carbon, from insignificant levels at 350 °C to >80  ± 2 at. % at 800 °C. Simultaneously, Pd surface signals decline sharply at high temperatures, indicating partial coverage by carbonaceous deposits or encapsulated Pd. The Zr signal initially increases (350–500 °C) due to oxidative segregation of ZrO_2_ on top of Pd–Zr, but decreases beyond 650 °C as carbon progressively shields the outermost layers.

**Figure 3:**
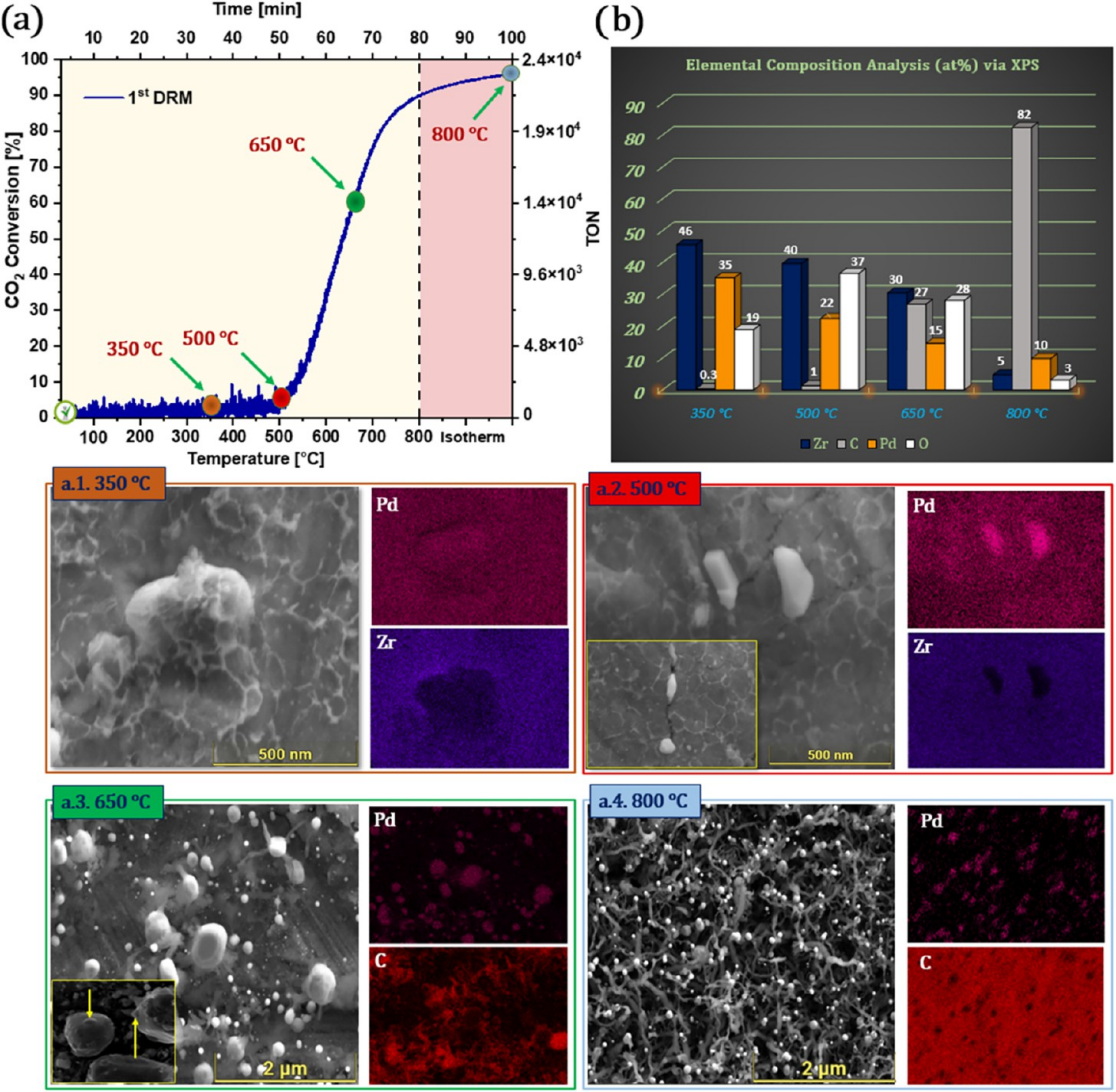
(a) Catalytic performance and surface evolution of initially intermetallic Pd/Zr during the first DRM cycle (CH_4_:CO_2_ = 1:1). The CO_2_ conversion profile is shown beside SEM images and EDX elemental maps at key temperatures: (a.1) 350 °C; (a.2) 500 °C; (a.3) 650 °C; (a.4) 800 °C. (b) Surface atomic composition (at. %) derived from XPS at each temperature. Reaction conditions: 50 mbar of CH_4_, 50 mbar of CO_2_, 977 mbar of He, linear temperature ramp (10 °C min^–1^) up to 800 °C, followed by isothermal reaction for 30 min.

These results reveal a highly dynamic Pd/Zr system during DRM, characterized by initial CO_2_-induced ZrO_2_ covering and PdZr decomposition/restructuring, associated with Pd particle exsolution and growth, and eventual carbon supersaturation via CH_4_-splitting on protruded metallic Pd, leading to Pd particle detachment and carbon filament formation via the tip-growth mechanism.^45^ This structural evolution is directly linked to the emerging catalytic activity, as the appearance of gas-phase-accessible Pd at intermediate temperatures coincides with the sharp onset of CO_2_ conversion. However, this process also appears to facilitate the onset of severe coking, reflecting an inherent trade-off between catalytic activity and coke accumulation.

As a tentative explanation, we propose a two-stage activation and restructuring pathway of the Pd/Zr catalyst under DRM conditions. During stage I (<500 °C), i.e., in the early activation stage, structural rearrangements remain largely confined to the near surface of the PdZr intermetallic. CO_2_, being more kinetically accessible than CH_4_ at these temperatures, preferentially oxidizes the outermost layers of the alloy, giving rise to a thin passivation shell of ZrO_2_. Within this layer, only a limited fraction of Pd is released, which emerges as small nanoparticles intimately anchored to the oxide surface. At this initial stage, these nanoparticles remain essentially carbon-free simply because methane activation is still limited, and carbon deposition has not yet commenced. At the same time, CO_2_ is readily activated at the metal–oxide interface. As a result, coking is essentially suppressed in this stage. During stage II (>500 °C), i.e., at higher temperatures, enhanced atomic mobility within the PdZr bulk drives more enhanced structural changes. Large Pd agglomerates form beneath the passivation layer and gradually protrude through cracks, yielding micron-sized domains at the surface. Simultaneously, the initially small Pd nanoparticles begin to sinter, resulting in a bimodal size distribution. These larger Pd entities accelerate methane activation, but their reduced interfacial contact with ZrO_2_ renders carbon removal less efficient. Consequently, the system shifts toward uncontrolled carbon filament growth, often accompanied by particle detachment and the formation of an interwoven carbon overlayer characteristic of tip-growth mechanisms.

To further resolve the correlation between DRM reactivity and surface carbon formation, we conducted a systematic series of partial conversions, stopping the reaction at five distinct CO_2_ conversion levels: 30, 40, 50, 60, and 100%. The resulting XPS spectra (Figure 4, left panel) reveal a clear sequence of increasing carbon signal intensity with increasing conversion, establishing a strong correlation between high conversion and coke buildup. At low conversions (30–40%), the surface remains relatively free of carbonaceous species, as indicated by weak C 1s signals and the absence of carbon deposits in SEM. Importantly, this part also corresponds to the absence (or undetectable small size) of detached Pd nanoparticles on the surface, preventing the onset of filamentous carbon growth. At ∼50% conversion, however, a structural threshold is reached: Pd begins to exsolve from the Pd–Zr matrix in the form of almost micrometer particles/clusters. These newly liberated Pd units provide the essential active sites required for the tip-growth mechanism of carbon filaments, explaining why carbon becomes detectable first at this stage. This trend intensifies markedly at 60% conversion, with SEM images displaying clear fibrous structures, and finishes in a highly coked surface at full (100% ± 1%) conversion, characterized by dense, network-like carbon fiber deposits and significantly suppressed surface detectability of the underlying catalyst substrate. Altogether, the XPS and SEM data clearly demonstrate that coke accumulation during DRM proceeds not linearly, but exhibits a threshold behavior that accelerates beyond ∼50% CO_2_ conversion. Particularly, operating below this threshold, i.e., maintaining DRM at sub-60% conversion, offers a practical pathway for preserving a clean, carbon-free catalyst surface, effectively avoiding the onset of deactivation and coking.

**Figure 4:**
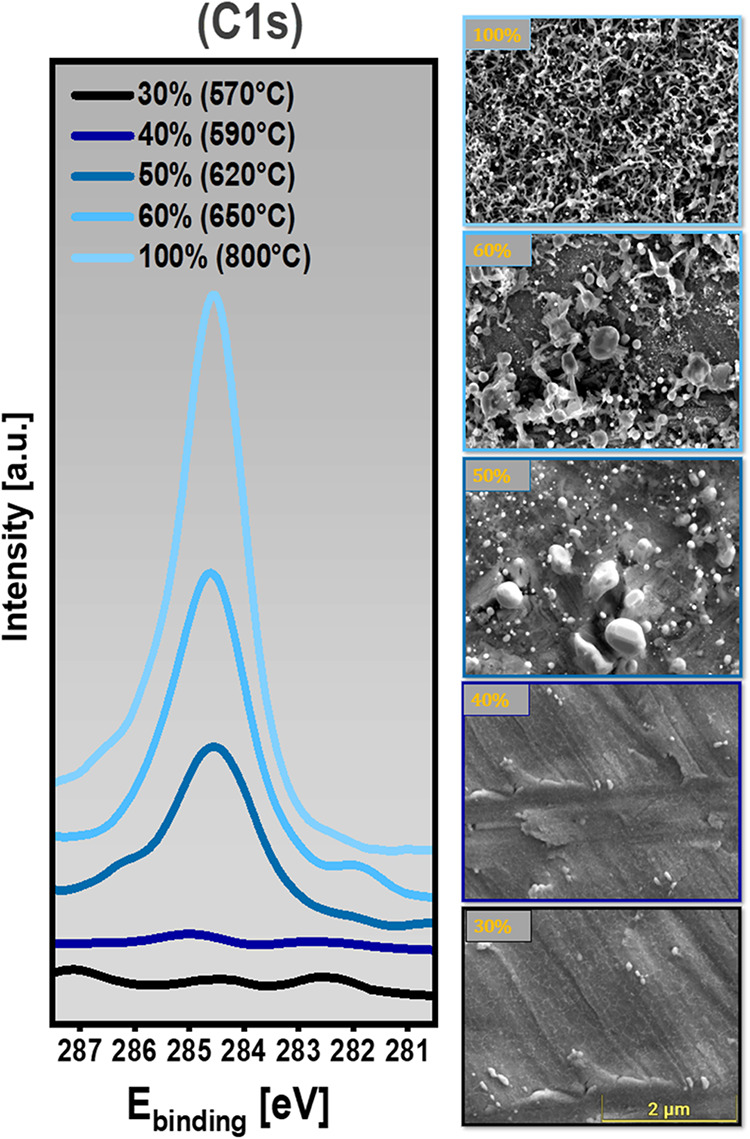
Evolution of surface carbon deposition and Pd particle/cluster exsolution with increasing CO_2_ conversion during DRM on Pd/Zr. Left: XPS C 1s spectra showed increasing carbon signal intensity from 30 to 100% CO_2_ conversion. Right: Corresponding SEM images at each conversion level (from bottom to top: 30, 40, 50, 60, 100%).

A closer examination of the catalyst at 650 °C (Figure 5) reveals significant morphological differences. The SEM image displays two distinct groups of Pd particles on the surface: many small particles (around 160 nm) and a second group of much larger particles exceeding 1.5 μm in diameter. This bimodal size distribution, shown in the histogram, indicates the ongoing restructuring of the Pd/Zr surface during the reaction conditions. The smaller particles can be understood as Pd entities forming near the surface in close interaction with the ZrO_2_ overlayer, while the much larger particles likely originate from subsurface Pd that accumulates beneath the passivating ZrO_2_ layer and subsequently emerges through cracks, as already observed at 500 °C (Figure 3a.2). This interpretation is consistent with the dynamic restructuring of the Pd/Zr system: near-surface Pd sites remain finely dispersed and well interfaced with ZrO_2_, whereas deeper-lying Pd migrates out and merges into large domains under reaction conditions. This structural variety is important because it suggests that different Pd particle sizes could interact differently in catalytic activity and carbon formation. Notably, the presence of the larger Pd domains aligns with the first visible carbon filaments seen in Figure 3a.3, supporting the connection between Pd accessibility and the start of coking.

**Figure 5:**
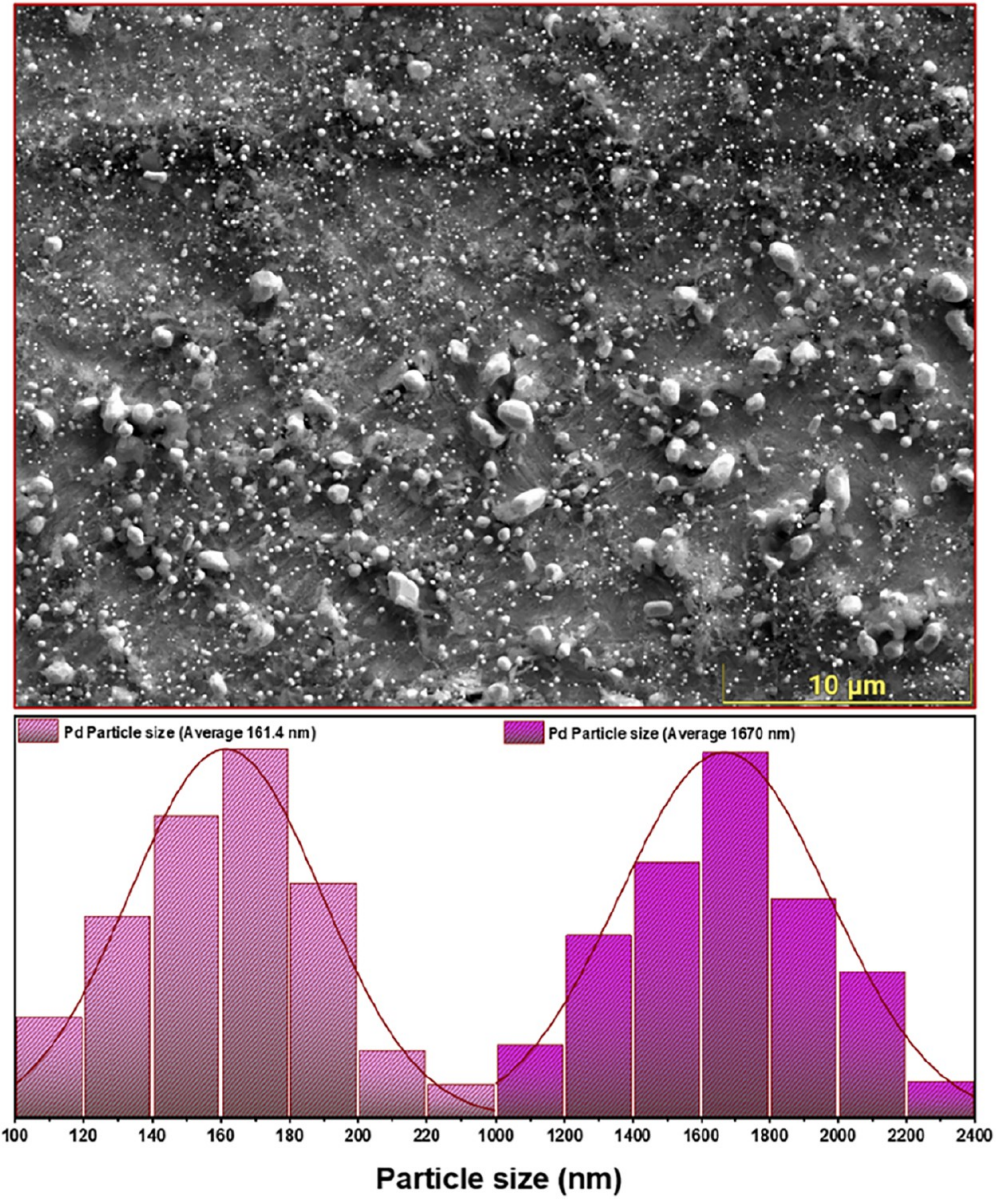
SEM image and particle size distribution of Pd/Zr after exposure to 650 °C during DRM. A statistical evaluation of more than 120 particles reveals an average size of 161 ± 3 nm for the small Pd population and 1670 ± 70 nm for the large Pd agglomerates.

By the end of the first DRM cycle at 800 °C, the Pd/Zr surface undergoes significant structural and chemical changes (Figure 6a). The SEM overview shows a dense network of carbon filaments, an obvious sign of severe coking. The corresponding particle size distribution (inset) shows a shift back to a unimodal profile with an average diameter of ∼160 nm. This apparent dominance of small Pd particles can be explained by the tip-growth mechanism of filamentous carbon: only Pd particles of this intermediate size (∼160 nm) are sufficiently mobile to detach from the surface and become incorporated at the tips of the growing carbon filaments. In contrast, larger Pd domains are unable to detach and instead remain buried beneath the thick carbon overlayer, rendering them undetectable in the postreaction size analysis. This change highlights the highly dynamic restructuring of the Pd phase during the reaction. The detailed SEM images (Figure 6b–d) offer clear insights into the interaction between Pd particles and carbon. In Figure 6b, individual Pd nanoparticles act as growth sites for carbon filaments, which extend in multiple directions like “arms” attached to the metal. The large enough Pd particles are frequently supersaturated with carbon to a degree that enables the nucleation of multiple filaments simultaneously. This explains the “triple-arm” morphology observed in several cases. Such multidirectional growth is not random but appears linked to specific crystallographic facets of the Pd surface, giving rise to the lobotomized impression of particles being penetrated by several filaments at once. The SEM-EDX analysis in Figure 6c confirms the direct role of Pd nanoparticles in filamentous carbon growth. Here, Pd particles serve as anchoring points for carbon structures spreading outward. EDX elemental maps (Figure 6c) show the distribution of Pd and carbon, confirming that these filaments originate specifically in Pd-rich areas. Moving to Figure 6d, closer inspection reveals distinct surface patches on the Pd particles, which are likely preferred sites for carbon filament growth. Additionally, a partially formed carbon “hump” (indicated by the green arrow) suggests an arrested or very early filament growth stage, probably because the reaction stopped before the filament could fully develop. Overall, these findings emphasize the intrinsic tendency of bulk-decomposed Pd/Zr catalysts to promote filamentous carbon growth. The surface features on Pd, possibly related to locally ordered Pd–C surface phases, e.g., at reconstructed facets, seem to provide favorable sites for filamentous carbon formation.

**Figure 6:**
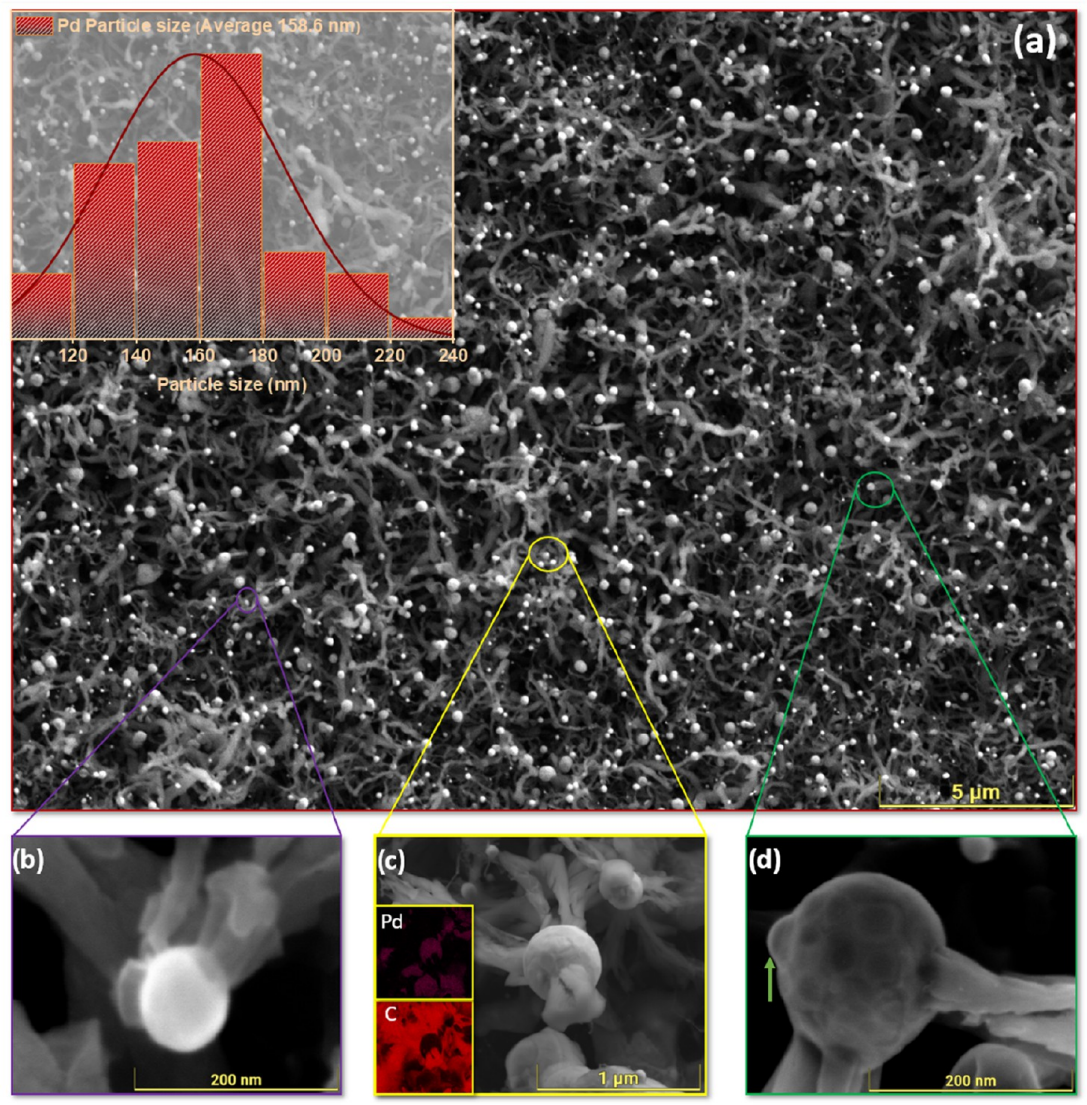
(a) SEM overview and particle size distribution of Pd/Zr after the DRM at 800 °C. (b–d) High-resolution SEM images highlighting Pd particles and associated carbon structures, with EDX elemental maps shown in the insets in (c). A statistical evaluation of more than *n* = 120 particles reveals an average size of 159 ± 2 nm for the Pd population.

The evolution of surface carbon during DRM is confirmed by Raman spectroscopy (Figure 7), which provides a complementary perspective with respect to the SEM and XPS analyses. At lower DRM temperatures (350 and 500 °C), the spectra remain dominated by oxide-related features, with characteristic bands of monoclinic ZrO_2_ (m-ZrO_2_) visible. This suggests that carbon deposition is insignificant in this stage, consistent with the limited conversion and the morphological observations shown in Figure 3a. A sharp change occurs at 650 °C. Here, distinct D (1348 cm^–1^) and G (1583 cm^–1^) bands emerge, indicative of disordered (D-band) and graphitic (G-band) carbon. This supports SEM evidence of early filamentous growth (yellow arrows, Figure 3a.3), indicating the onset of coking. The increased D/G intensity ratio further highlights the formation of highly defective, amorphous carbon in an initial stage of deactivation. At 800 °C, the carbon signatures increase significantly, with pronounced D and G bands, along with their overtones D′ (1614 cm^–1^), G′ (2695 cm^–1^), and a weak 2935 cm^–1^ feature. This reflects massive carbon buildup, in line with the complementary results observed by SEM (Figure 6a). Notably, the oxide features decrease, suggesting progressive surface shielding by the growing carbon top layer. This spectroscopic insight confirms the direct relationship between increasing DRM temperature, catalytic activity, and the degree of coking, further illustrating the challenge of balancing high conversion and coke resistance in Pd/Zr systems. SEM, Raman, and XPS collectively indicate that the carbon formed on Pd/Zr consists predominantly of filamentous, turbostratic, and sp^2^-rich structures. Filament nucleation begins near ∼50% CO_2_ conversion, while full DRM conditions yield a dense nanofiber network. Fully graphitic encapsulating layers were not observed. The filamentous character of the coke explains the tip-growth mechanism and the strong dependence of the regeneration ability on the presence of accessible Pd–ZrO_2_ phase boundaries.

**Figure 7:**
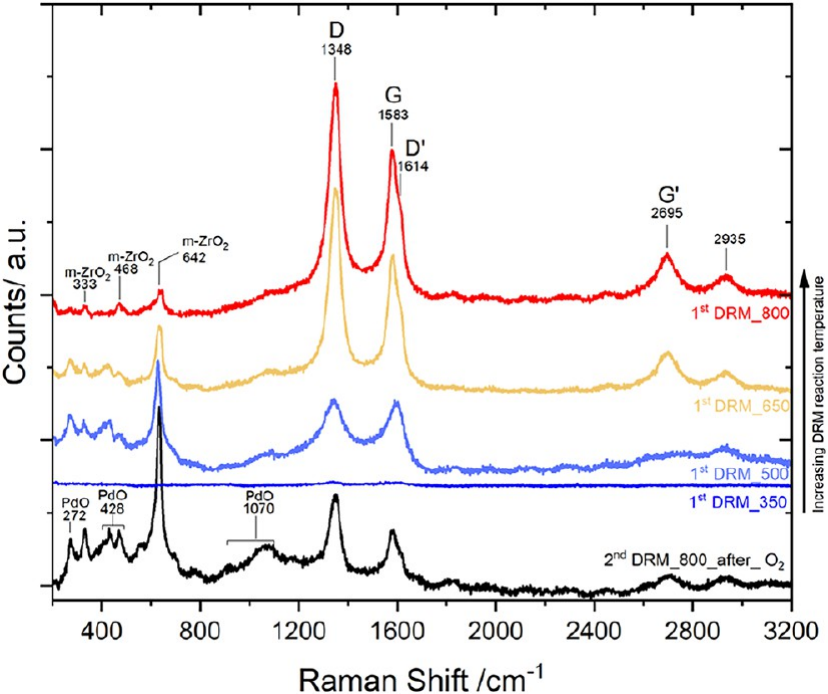
Raman spectra of the initially intermetallic Pd/Zr catalyst during DRM at increasing reaction temperatures (350, 500, 650, and 800 °C) and after regeneration, followed by a second DRM cycle.

So far, the initial DRM studies strongly link high Pd activity and conversion to widespread coking, but the main sources of this carbon deposition remain to be clarified. In previous studies, CH_4_ decomposition was identified as the primary source of coke.^1,14,46^ Yet, considering the high conversion levels achieved within our batch reactor configuration, whereby the CO and H_2_ levels constantly increase and eventually replace the educts CO_2_ and CH_4_ in the gas phase, it appears necessary to ask to what extent the formed syngas contributes to the accelerated high-temperature coking. In order to quantify this contribution, the Pd/Zr catalyst was directly exposed to CO and H_2_ under DRM-analogous conditions. The following section investigates whether these products can independently induce carbon formation and sheds light on alternative pathways of coke buildup with respect to methane decomposition.

#### 3.2.3 Syngas (CO + H_2_) Exposure Reveals an Alternative Route for Carbon Deposition

Figure 8 shows the results of exposing the initially intermetallic Pd/Zr catalyst to CO + H_2_ (syngas) instead of CH_4_ + CO_2_ to explore the role of DRM products in the coking behavior. In Figure 8a, the CH_4_ pressure profile displays a clear initial rise as methanation occurs in the low-temperature region: CO + 3H_2_ → CH_4_ + H_2_O. The steep increase of the methane signal toward a maximum at about 350 °C reflects rapid CO hydrogenation below 350 °C and the establishment of the methanation reaction equilibrium. However, as the temperature continues to increase, a turning point appears, and the CH_4_ partial pressure steadily decreases. This decline indicates that the thermodynamic reversal of methanation, i.e., the steam reforming equilibrium (CH_4_ + H_2_O ⇌ CO + 3H_2_), becomes prevalent. At the same time, the possible formation of CO_2_ suggests that the reaction network is more complex, resembling a “bi-reforming” environment. Figure 8b shows the morphology after CO + H_2_ exposure. The SEM image reveals significant carbon buildup along cracks and defects, again forming filamentous carbon structures. The inset highlights nanoscale Pd particles connected to the tips of the carbon filaments. EDX mapping confirms widespread carbon coverage, along with dispersed Pd, Zr, and oxygen signals. Notably, the carbon distribution in the EDX maps appears less dense than after exposure to pure CH_4_ (Figure 2b), indicating a lower carbon yield, which aligns with the milder coking behavior of syngas, and especially of the CH_4_–CO–CO_2_–H_2_O reaction mixture formed from syngas at intermediate temperatures, as compared to pure CH_4_. Figure 8c supports these observations, showing that although surface carbon deposits are present, they appear to a lesser extent than after pure CH_4_ exposure or a full DRM cycle. Also, the Zr 3d and O 1s signals are still detectable, indicating weaker shielding/partial spectroscopic surface accessibility. For quantitative insight, Figure 9 provides an atomic percentage (atom %) analysis derived from the XPS data. The results suggest that syngas (i.e., the DRM product mixture) can act as a carbon source capable of causing coke formation even without CH_4_ or CO_2_ in the initial feed. Moreover, they clearly show that the amount of carbon formed during the first DRM cycle is notably higher than that observed in either pure CH_4_ or CO + H_2_ exposures alone. We suggest that during DRM, all three possible carbon formation pathways: methane decomposition CH_4_ → C + 2 H_2_, inverse Boudouard reaction 2 CO → C + CO_2_, and inverse water gas reaction CO + H_2_ → C + H_2_O are simultaneously active and add up to a maximum coking level. Conversely, this highlights an important aspect of the DRM reaction: at high CO_2_ conversions, these synergistic processes contribute to increased carbon deposition, as elevated partial pressures of CO and H_2_ (relative to those of CO_2_ and H_2_O) rather promote the above-mentioned reverse reactions on the catalyst surface, thereby suppressing the most important carbon clean-off routes. The effect of shifted equilibria offers a compelling explanation for the significantly enhanced carbon signals seen at high conversions in Figure 4

**Figure 8:**
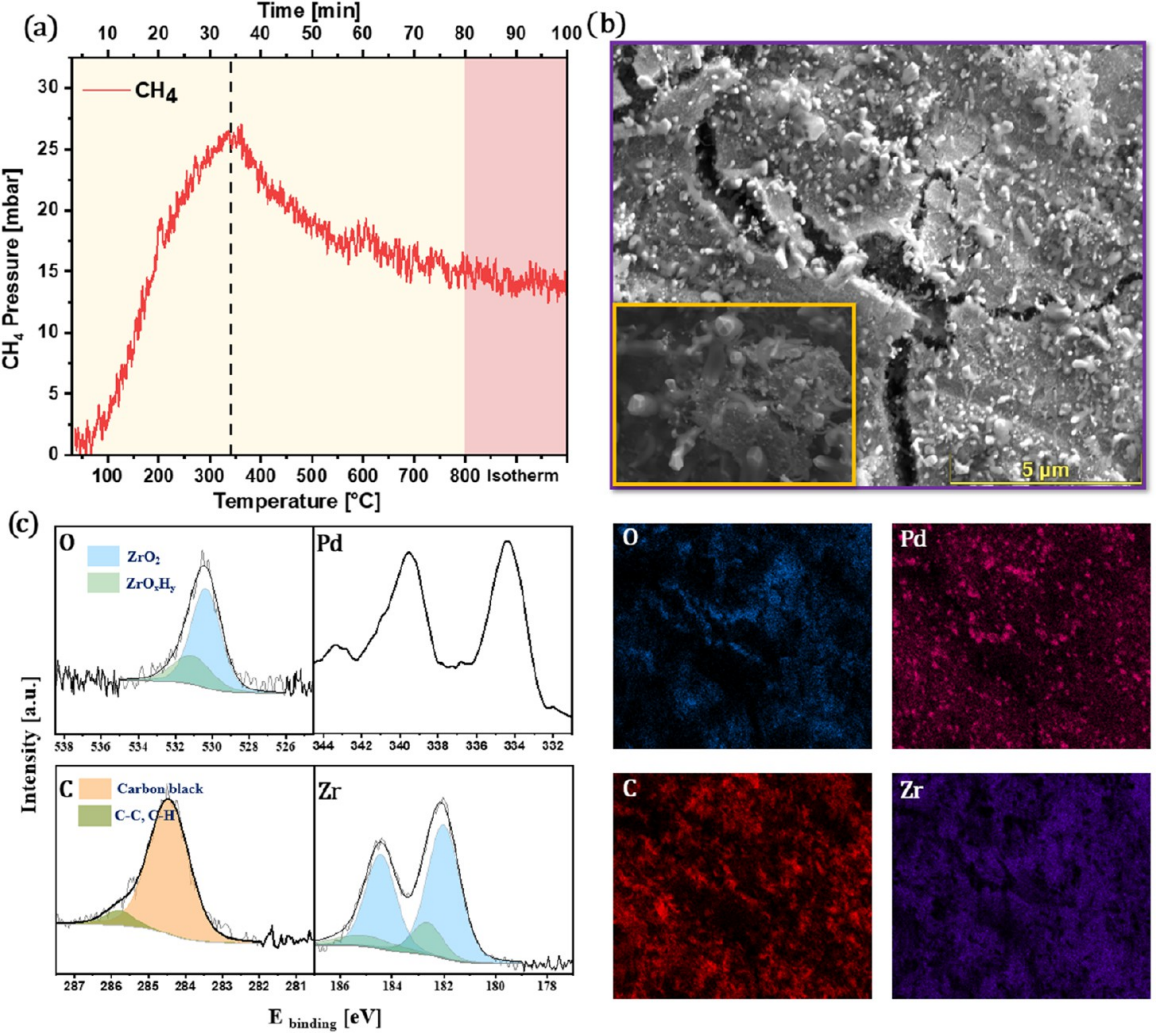
Panel (a) CH_4_ pressure profile during Pd/Zr exposure to CO + H_2_ (syngas); panel (b) SEM image and EDX maps after syngas exposure; panel (c) XPS survey spectra of the surface after temperature-programmed reaction in syngas. Reaction conditions: 50 mbar of CO + 50 mbar of H_2_, linear temperature ramp (10 °C min^–1^) up to 800 °C, followed by isothermal reaction for 30 min.

**Figure 9:**
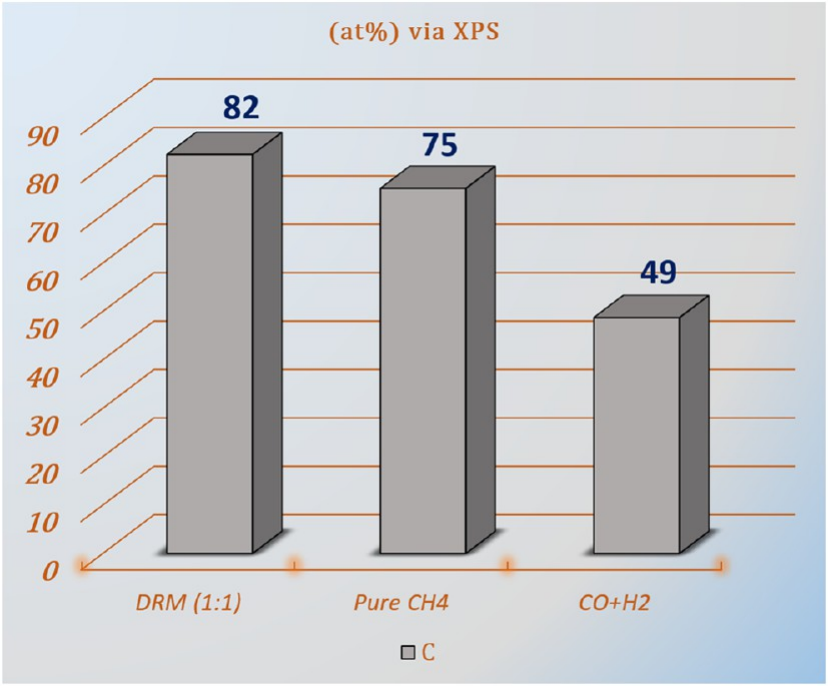
Surface carbon content (at.%) measured by XPS after exposure to DRM (1:1 CO_2_:CH_4_), pure CH_4_, and CO + H_2_ (syngas).

The prior results highlight the intrinsic challenge of balancing high catalytic activity with coke resistance in Pd/Zr systems. Both direct methane decomposition and secondary interactions with syngas (CO + H_2_), while suppressing carbon clean-off routes linked to the presence of sufficient H_2_O and CO_2_, contribute to enhancing coking. To address this, a reasonable strategy is to change the reaction environment, e.g., by increasing the CO_2_/CH_4_ ratio. In the following section, we explore how a 2:1 CO_2_:CH_4_ feed impacts the coking behavior and overall catalytic performance of Pd/Zr during DRM.

#### 3.2.4 Suppression of Coking by Feed Tuning (2:1 CO_2_:CH_4_)

Figure 10 presents an effective case for the strategic use of CO_2_-enriched feeds to mitigate carbon deposition during DRM over Pd/Zr catalysts. In Figure 10a, the CH_4_ conversion profile obtained at a 2:1 CO_2_:CH_4_ ratio reaches nearly 100 ± 1%, comparable to the stoichiometric 1:1 ratio, indicating that catalytic activity remains uncompromised despite the changed feed composition. However, the impact on coking is deep. Figure 10b compares the surface carbon spectra (C 1s XPS) between the two feed ratios. While both conditions achieve full CH_4_ conversion, the catalyst exposed to the CO_2_-enriched feed builds up significantly less carbon on its surface. The inset quantifies this difference: the carbon atomic percentage drops dramatically from 82 ± 2% (1:1) to 19 ± 2% (2:1). This reduction in surface carbon indicates that excess CO_2_, beyond its role as a reactant, acts as an in situ removing agent, likely promoting the reverse Boudouard reaction (CO_2_ + C → 2CO), thereby continuously removing deposited carbon during operation. The corresponding CO MS signal under CO_2_-rich conditions is shown (Figure S3). Notably, the system maintains high catalytic turnover without crossing into the coking zone, confirming that oxidant-rich DRM feeds represent an effective means to reduce carbon deposition without sacrificing conversion.

**Figure 10:**
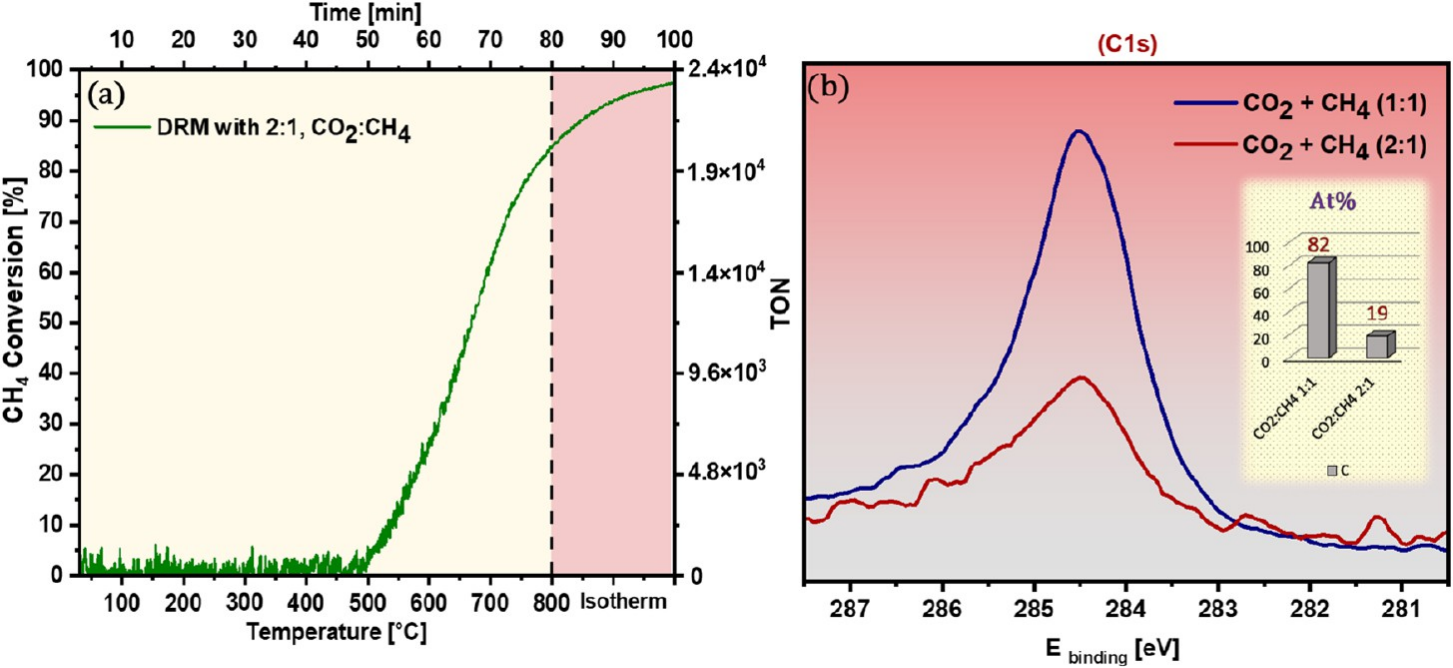
(a) CH_4_ conversion profile during DRM with a CO_2_:CH_4_ feed ratio of 2:1; (b) C 1s XPS spectra comparing surface carbon buildup after DRM with 1:1 and 2:1 feed ratios (The inset bar graph quantifies the carbon atomic percentage). Reaction conditions: 100 mbar of CO_2_ + 50 mbar of CH_4_, linear temperature ramp (10 °C min^–1^) up to 800 °C, followed by isothermal reaction for 30 min.

Despite the tuning of feed composition, coking remains an important risk in dry reforming systems. To assess the practicality of the Pd/Zr catalyst, one must look beyond prevention strategies and face the questions of structure and activity recovery.

#### 3.2.5 Regeneration Behavior and the Role of Phase Boundaries

To evaluate not only the activity but also the recoverability of the fully coked Pd/Zr catalyst under DRM conditions, a two-cycle dry reforming experiment (1:1 condition with respect to CO_2_ and CH_4_) was performed (Figure 11a). As expected, the first DRM cycle delivered full CO_2_ conversion (∼100% ± 1%). However, in the second successive DRM cycle, catalytic activity dropped significantly to below 50% ± 1%. This decline is a clear indication of carbon accumulation, as evidenced by the intense carbon signal in the postreaction XPS spectra (Figure 11b). Yet, the catalyst was not fully deactivated; some residual activity persisted, pointing toward hidden active domains, likely buried Pd/Zr interfacial boundaries, that survived the coking stage and continued to supply the reforming process. In trying to regenerate the catalyst, a treatment in pure CO_2_ was applied (Figure 11c, curve 1). Unexpectedly, this relatively gentle oxidative environment failed to remove deposited carbon. We suggest that this inability is linked to the virtual absence, or at least the lack of gas-phase accessibility of metal oxide phase boundaries, which are vital sites for CO_2_ dissociation and reactive oxygen formation. Without active interfaces, the carbon deposits remained inert toward CO_2_. This behavior contrasts with findings from our previous study on the analogous Ni/Zr system,^47^ where active phase boundaries between Ni and ZrO_2_ enabled successful regeneration in CO_2_. Moreover, CO_2_ dissociation is intrinsically possible already on the metallic Ni surface itself, in contrast to Pd surfaces, which do not dissociate CO_2_ due to exceedingly high kinetic barriers.^14^ Therefore, the blocked regeneration in CO_2_ results most likely from the combination of suppressed metal surface oxidation and a lack of remaining active metal oxide phase boundary sites in the fully coked Pd/Zr system. In addition to the limited availability of surface Pd–ZrO_2_ phase boundaries, Pd is intrinsically disadvantaged in CO_2_ dissociation due to its very late, high-energy transition state, making CO_2_ activation on Pd surfaces kinetically unfavorable compared to Ni. Combined with Pd’s highly efficient methane activation, this imbalance leads to rapid carbon accumulation while suppressing CO_2_-mediated carbon clean-off. Once the Pd–ZrO_2_ phase boundaries become blocked by this carbon, the DRM process, which fundamentally relies on these PB sites, comes to a complete halt.^48^ This view is further confirmed by the SEM images in Figure 6, which reveal extensive carbon overgrowth and substrate shielding, both effectively trapping the detached Pd particles in carbon shells and eliminating potential direct gas-phase contact with the partially oxidized PdZr/ZrO_2_ support underneath, thus breaking the critical interfacial architecture required for phase-boundary-mediated carbon oxidation toward CO.

**Figure 11:**
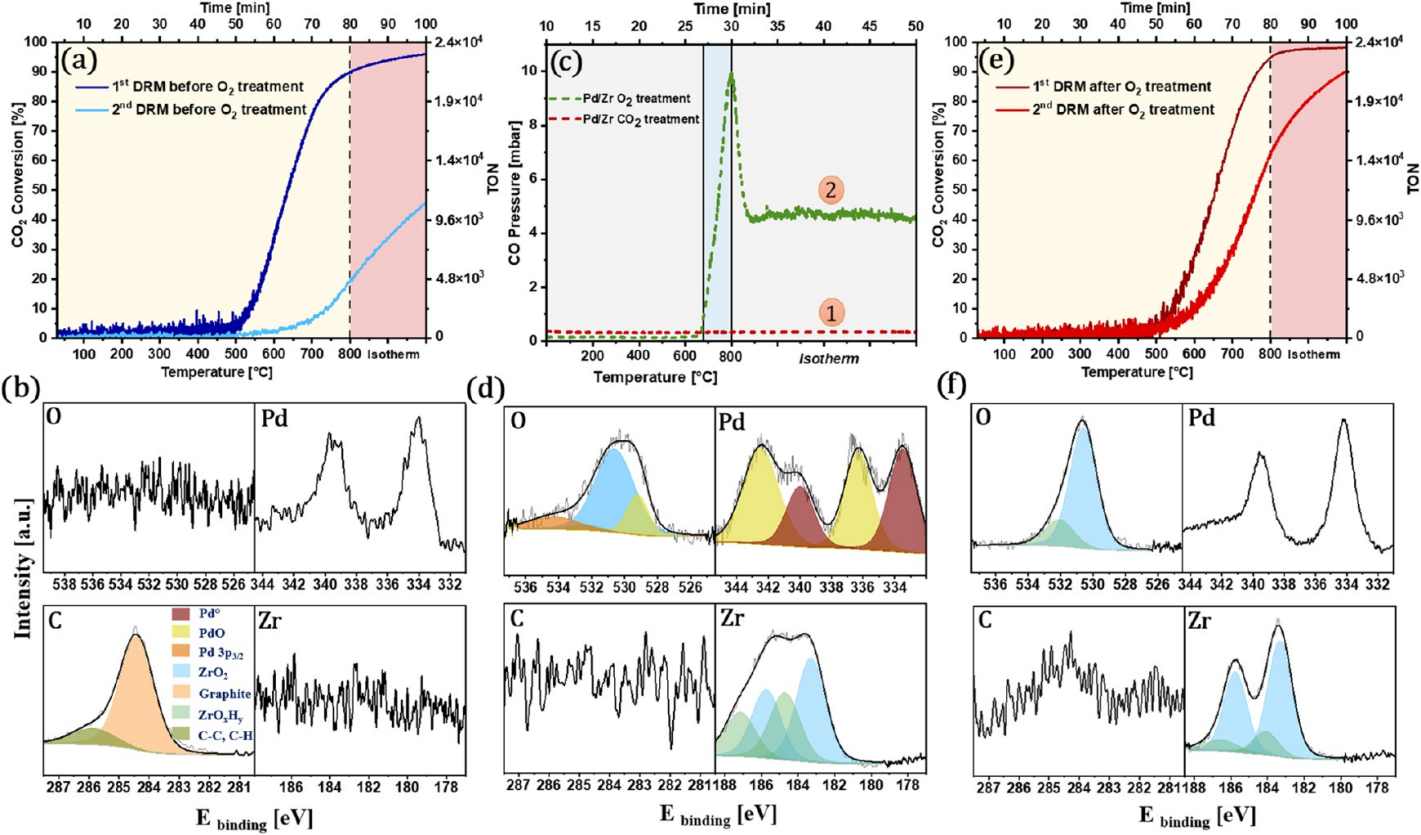
Regeneration behavior of Pd/Zr during DRM cycling. (a) CO_2_ conversion over two successive DRM runs without previous regeneration, starting from the initial intermetallic state; Reaction conditions: 50 mbar CH_4_ + 50 mbar CO_2_, 977 mbar He, linear temperature ramp (10 °C min^–1^) up to 800 °C, followed by isothermal reaction for 30 min. (b) XPS spectra after coking-induced deactivation. (c) Regeneration profiles using CO_2_ (unsuccessful (1)) and O_2_ (effective (2)); reaction conditions: 100 mbar CO_2_/O_2_ (heating rate 38 °C min^–1^) (d) XPS after O_2_ treatment; (e) recovery of conversion after O_2_ regeneration; (f) XPS after second postregeneration DRM.

To overcome this, an O_2_ treatment was performed post-DRM (Figure 11c, curve 2). The oxygen environment immediately caused a sharp CO formation signal, indicating gasification of carbon species via combustion. Postregeneration XPS spectra (Figure 11d) confirm almost complete removal of surface carbon and the reestablishment of the O 1s and Zr 3d signals. Notably, the subsequently recorded Pd 3d spectrum revealed partially oxidized palladium species, indicating that a part of the metal became oxidized during the O_2_ treatment. In due course, this intermediate partial oxidation of Pd played a pivotal role. When the O_2_-regenerated catalyst was reintroduced into the next DRM cycle (Figure 11e), the system showed not only fully restored “first-cycle” performance, but also strongly improved coking properties during the second cycle, thereby maintaining ∼90% ± 1% conversion throughout the reaction. Furthermore, repeated reaction–regeneration cycling demonstrates that the Pd–Zr catalyst maintains its high activity: after four consecutive DRM → O_2_ regeneration → DRM cycles, the CO_2_ conversion profiles consistently reach ∼95–100% within the isothermal period, confirming the excellent regeneration ability and operational robustness of the Pd–Zr system (Figure S4). The slight variations observed in the CO_2_ conversion profiles before reaching the highest conversion levels across successive cycles are likely caused by progressive Pd particle sintering, which arises due to the repeated transition of the agglomerated oxidized state and the more ineffective Pd redispersion after the respective DRM tests.

This distinct improvement suggests that in situ reduction of PdO species back to Pd metal under DRM conditions most likely yields a modified metallic Pd state enriched with subsurface/bulk-dissolved oxygen species,^36^ which subtly shifts the electronic and structural properties of Pd toward enhanced coking resilience. We note that Pd redox chemistry is intrinsically complex, and several studies have shown that the PdO → Pd transition is typically accompanied by particle growth and morphology changes. In particular, Farrauto et al.^49^ and Chen et al.^50^ report that the reduction of PdO often leads to sintered metallic Pd clusters with modified surface reactivity. In the context of our Pd/Zr system, it is therefore reasonable to assume that not only Pd accessibility and phase-boundary formation but also the transient presence of PdO and the associated PdO/Pd transformations contribute to the observed changes in coking behavior before and after oxygen regeneration. Although our present data set does not allow us to quantitatively resolve PdO at all stages, the Raman trends and the evolution of Pd particle morphology (Figure 12c) are consistent with a scenario where Pd redox and particle size effects act in concert with phase-boundary changes. To further resolve the structural and compositional evolution during the postregeneration DRM cycle, we also performed intermediate quenching experiments at 350, 500, and 650 °C in the first DRM cycle after O_2_ treatment. The corresponding SEM-EDX images and CO_2_ conversion profiles are shown in Figure S5. Finally, XPS characterization after the second postregeneration DRM cycle (Figure 11f) shows minimized carbon formation along with hardly suppressed Pd and Zr signals, further indicating the origin of sustained catalytic activity.

**Figure 12:**
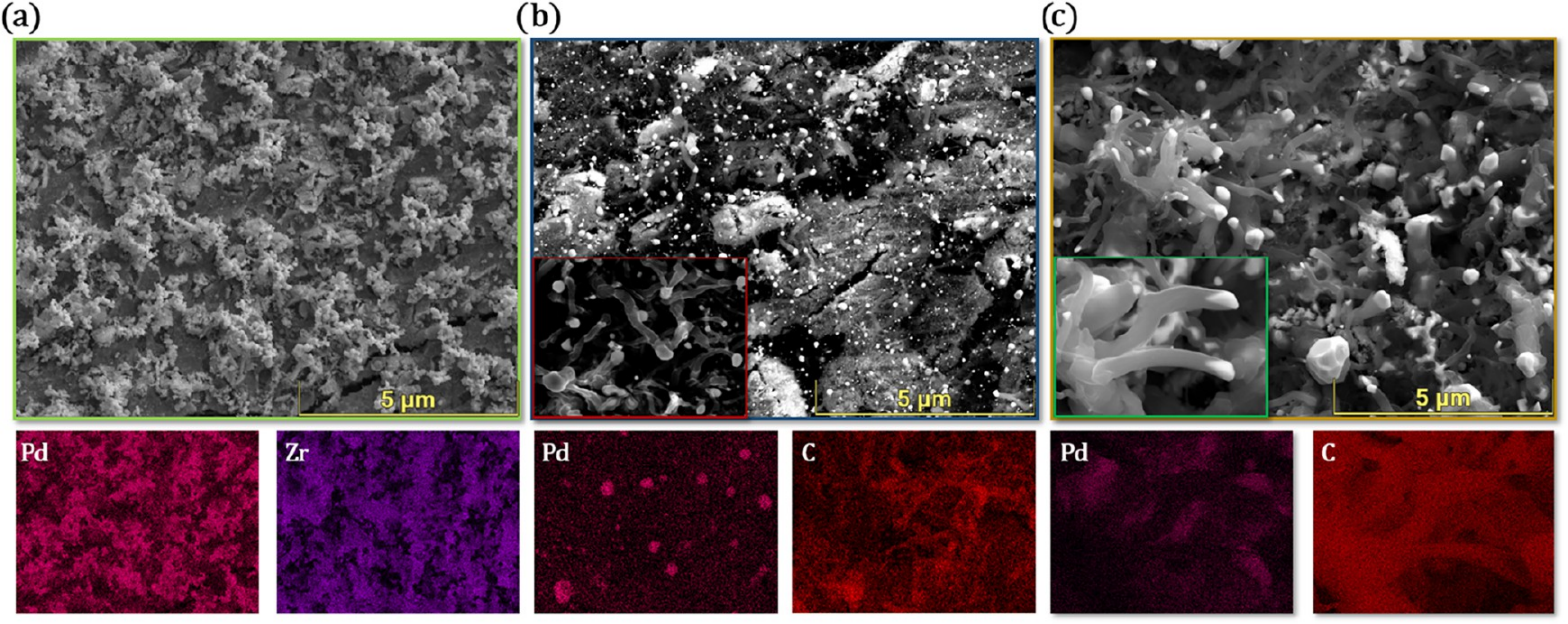
Morphological evolution of the initially fully coked Pd/Zr catalyst (see Figure 6) during regeneration and subsequent DRM cycles: (a) Clean catalyst surface after O_2_ treatment; (b) after first postregeneration DRM cycle; (c) after second postregeneration DRM cycle.

To visualize the structural and compositional results of the regeneration strategy, Figure 12 provides a comparative set of SEM and EDX images across key stages of the regeneration procedure. Figure 12a shows the Pd/Zr surface immediately after O_2_ treatment, where the extensive carbon deposits previously observed in Figure 6 have been eliminated. The surface state appears clean and also structurally reestablished. After O_2_ regeneration, Pd appears heavily agglomerated into irregular clusters, preventing a reliable determination of a particle size distribution. Nevertheless, SEM contrast clearly confirms significant Pd coalescence triggered by oxidative treatment. And reemerging ZrO_2_ domains, providing strong evidence for successful regeneration. In Figure 12b, which shows the catalyst surface following the first DRM cycle after O_2_ regeneration, the difference is outstanding. While carbon reappears, its morphology is more dispersed and far less dense than that of the filamentous networks of Figure 6. EDX maps support this observation, revealing a less intense carbon signal. Figure 12c highlights the surface state after the second DRM cycle postregeneration. Although carbon deposition has increased slightly relative to the first post-O_2_ cycle, it remains significantly milder than in the preregeneration state. The Pd signal is visible across much of the surface, and the filamentous carbon shows lower density and appears less tangled. These observations confirm a sustained improvement of catalyst resistance to deactivation across continuous reaction cycles.

In summary, the regeneration results highlight the vital role of oxidative treatments in O_2_ for reactivating Pd/Zr catalysts that experienced significant coking. The failure of CO_2_ as a regeneration oxidant, due to a lack of accessible metal–oxide phase boundary sites and the intrinsically low activity of the Pd surface toward CO_2_, sharply contrasts with the full restoration achieved through O_2_ exposure. Importantly, the postregeneration performance not only approaches the initial catalytic activity but also improves coking resistance in further DRM cycles. This behavior suggests a possible modification of the metallic Pd phase, most likely driven by subsurface/dissolved oxygen species, which provide both renewed accessibility and altered electronic properties. Although Pd/Zr does not possess the inherent recyclability with CO_2_ observed on the previously studied Ni/Zr system,^47^ strategic regeneration with O_2_ effectively restores the catalytic surface and maintains high performance over multiple cycles. While the present section established the regenerative power of O_2_ in restoring the heavily coked Pd/Zr system, it also exposed a key weakness: the inability of CO_2_ to oxidize deeply carbon-coated surfaces lacking a metal–oxide phase boundary. However, this limitation raised a fascinating possibility: what if the extent of carbon deposition is inherently lower, such as if the catalyst is only partially deactivated? Would CO_2_ then be sufficient to restore the activity?

#### 3.2.6 Regeneration in CO_2_ after DRM under CO_2_-Rich Conditions and after Reaction Quenching at Partial Conversion

One of the most surprising aspects of the Pd/Zr system emerges when regeneration experiments are conducted under milder, deliberately limited coking conditions. After confirming that CO_2_ alone cannot restore a fully deactivated, carbon-shielded catalyst, we shifted to conditions where carbon buildup was inherently lower, either due to CO_2_-enriched DRM feeds or by choosing partial conversion states. Figure 13a shows the regeneration profile following DRM with a 2:1 CO_2_:CH_4_ ratio. Unlike the heavily coked 1:1 case (Figure 11c), the CO signal increases noticeably with CO_2_ exposure, indicating active gasification of the surface carbon. XPS supports this observation (Figure 13b): although not all carbon can be removed, the C 1s intensity is diminished by almost 50%, as shown in the atomic percentage chart (inset). This partial decoking highlights a key mechanistic insight: when overall coking is reduced, a part of the metal–oxide phase boundaries remains accessible for CO_2_ activation and surface carbon clean-off. Even in the Pd/Zr system, which lacks the strong redox interplay seen in Ni/Zr, the correct feed composition creates a window in which CO_2_-based regeneration becomes feasible. The second experiment explores this concept further. Instead of driving the catalyst to full conversion, we intentionally stopped the first DRM cycle at about 50 ± 1% CO_2_ conversion. As previously noted, lower conversion results in less carbon buildup (Figure 4). After the CO_2_ treatment, the outcome was even more obvious (Figure 13c). The related XPS data (Figure 13d) show a significant decrease in the amount of surface carbon, again quantified in the inset. The deduced picture appears rather clear: moderate coke deposition again preserves active metal oxide phase boundaries, rendering also CO_2_ as a potentially practical oxidant for regeneration even in Pd-based systems, provided that the degree of coking is not excessively high. This insight establishes a direct connection between feed tuning, coking behavior, and regenerative pathways, also illustrating a potentially useful strategy: to limit CO_2_ conversion levels and use excess CO_2_ for limiting carbon deposition, in order to eventually facilitate CO_2_-based cleaning cycles without the need for more aggressive oxidative treatments.

**Figure 13:**
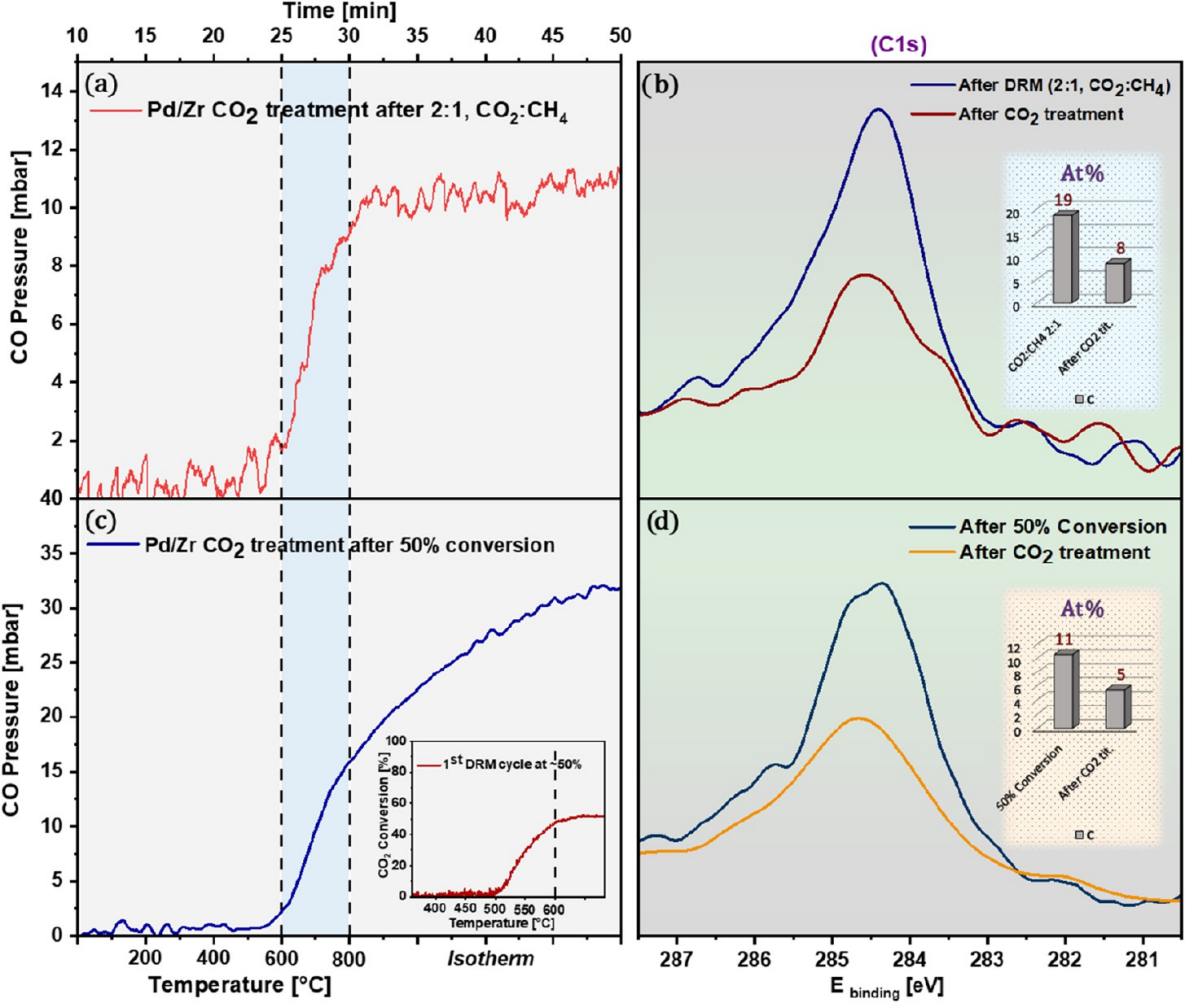
CO_2_-based regeneration under initially reduced coking conditions. (a) CO profile during CO_2_ treatment after a DRM cycle in a 2:1 CO_2_:CH_4_ feed. (b) C 1s spectra comparing the coked state and post-treatment surface. (c) CO profile after CO_2_ treatment of a catalyst quenched at ∼50% conversion reached at 600 °C. (d) Corresponding XPS before and after regeneration. Reaction conditions: 100 mbar of CO_2_, heating rate 38 °C min^–1^.

## 4 Conclusions

The present study establishes a detailed mechanistic framework for understanding how an initially intermetallic Pd/Zr catalyst develops under methane dry reforming conditions, which main coke deposition routes prevail on it, and how its final carbon balance can be managed through operational and regeneration strategies. By combining precisely controlled reaction environments with surface analysis, we were able to pinpoint multiple routes of carbon formation and identify the conditions that dictate whether carbon removal by CO_2_ is feasible or fails. A unifying picture emerges in which coking is not a simple byproduct of methane activation but the result of two converging effects, CH_4_ decomposition and the secondary influence of the produced CO/H_2_, whose combined effects intensify sharply at high CO_2_ conversions. Once carbon formation exceeds a critical threshold, the activated Pd/Zr surface becomes encapsulated, metal–oxide phase boundaries are covered, and CO_2_-assisted regeneration is no longer accessible. This contrasts with previous results on the analogous Ni/Zr system and highlights the crucial role of the interfacial sites even more: while Ni-based catalysts can sustain CO_2_-based regeneration even after heavy coking, heavily coked Pd/Zr requires oxidative treatments in O_2_ to fully restore the initial activity. Yet, our findings also reveal opportunities for the deliberate control of carbon coverage, involving the use of CO_2_-rich feeds or partial conversion. On moderately coked states of the catalyst, CO_2_ can reemerge as an effective and selective but also more gentle decoking agent. This interdependence between operating conditions, coking dynamics, and regeneration chemistry defines the practical trade-off between maximizing conversion and maintaining long-term catalyst health. Beyond feed tuning and regeneration protocol design, our findings also suggest that Pd oxidation state and particle size evolution (PdO ↔ Pd) are likely to play a nontrivial role in controlling coking in Pd–Zr systems. Tailoring Pd redox behavior via synthesis or pretreatment strategies may thus offer an additional lever for designing coke-resilient Pd-based DRM catalysts. Future studies using dedicated in situ redox-sensitive probes will be required to disentangle these contributions in more detail.

Scheme 1 captures the essence of the Pd/Zr catalytic cycle: the pathways into carbon formation, the points of no return where CO_2_ regeneration fails, and the operational windows where both high activity and coke control can coexist. Finally, the lessons to be learned from the initially intermetallic Pd/Zr model system extend beyond a single catalyst formulation. They highlight that the frequently used statement that “noble metal DRM catalysts are intrinsically more coke resistant” may be plain wrong, especially in the case of Pd. Yet, they can be engineered to operate within a “safe” carbon deposition region through deliberate feed tuning and regeneration design. Such strategies, grounded in basic mechanistic understanding, are critical for advancing DRM toward industrial relevance while achieving high conversion with a sustainable catalyst performance.

**Scheme 1:**
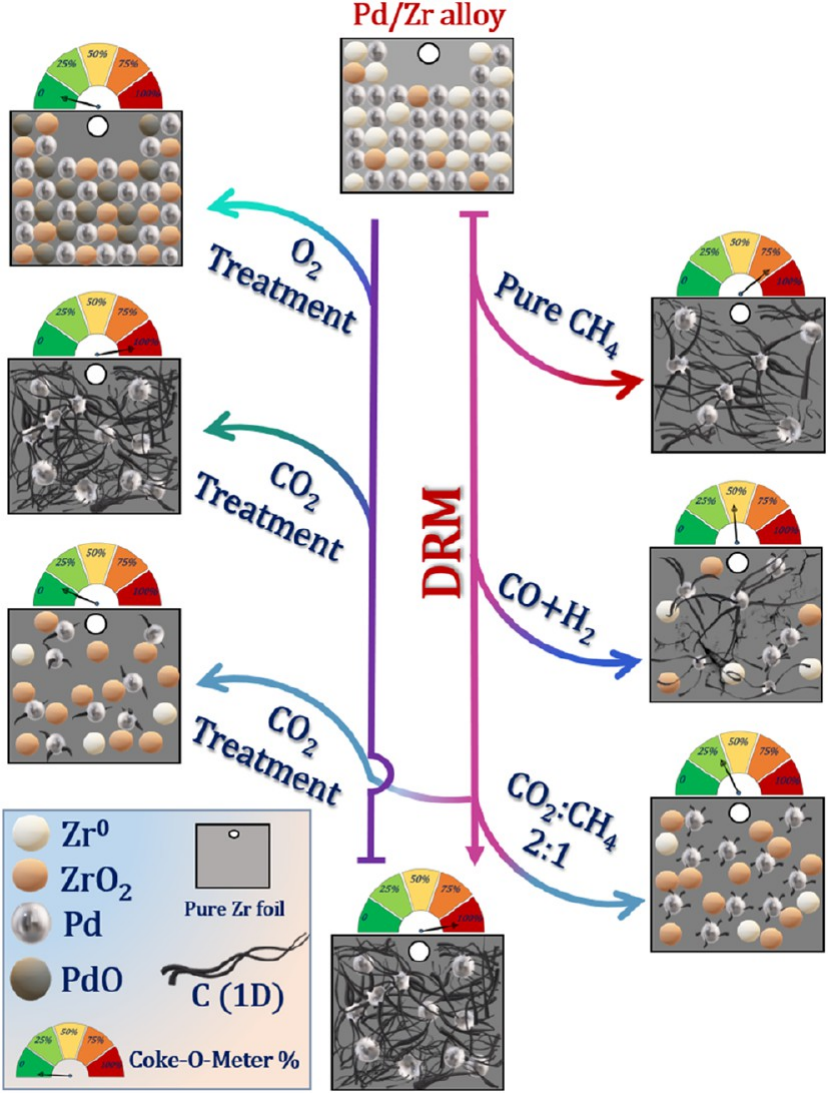
Schematic Survey of the Pd/Zr Catalyst Behavior and the Corresponding Coking and Regeneration Outcomes upon Applying Different Feed Compositions and Regeneration Strategies (Pure CH_4_, Syngas, DRM in 1:1 and 2:1 CO_2_:CH_4_)

## Supplementary Material



## Data Availability

Data will be made available on a repository under doi: 10.48323/vyjdp-50017

## Notes

Calculation of TOF and TON; CO desorption profiles after O_2_ regeneration; SEM images of the as-deposited Pd/Zr surface; CO_2_ conversion profiles after O_2_ treatment; additional CO mass spectrometer profiles; and CO_2_ conversion profiles for four DRM profiles with intermittent O_2_ regeneration (PDF)
