# Supplementary material for: To Coke or Not to Coke: When Pd Is Not Noble Anymore under Methane Dry Reforming Conditions
Source: ACS Catal. 2025 Dec 8;15(24):20980–96. doi: 10.1021/acscatal.5c07296 (PMC12723670; doi:10.1021/acscatal.5c07296)
Supplement: Supplementary file 1 [file cs5c07296_si_001.pdf]

To coke or not to coke: When Pd is not noble anymore under methane dry reforming conditions

Mahdi Hosseinpour,[1] Thomas F. Winterstein, [1] Clivia Hejny,[2] Marc Heggen,[3] Bernhard Klötzer,[1] Simon Penner[1]*

[1]*Institute of Physical Chemistry, University of Innsbruck, Innrain 52c, 6020 Innsbruck (Austria)*

[2]*Institute of Mineralogy and Petrography, University of Innsbruck, Innrain 52f, 6020 Innsbruck (Austria)*

[3] *Ernst Ruska-Centre for Microscopy and Spectroscopy with Electrons, Forschungszentrum Jülich GmbH, Leo-Brandt-Str. 1, D-52428 Jülich, Germany*

Corresponding author: Simon Penner*, simon.penner@uibk.ac.at, +4351250758003

Keywords: Dry reforming of methane, Palladium–zirconium catalysts, Catalyst regeneration, Metal–oxide phase boundary, Carbon deposition, tip-growth mechanism

Equation S1:

1.66×10^{15} Pd surface atoms cm^{-2}

1.66×10^{15} cm$^{-2} \times 7.2$ cm$^{2} = 1.2 \times 10^{16} \rightarrow$ total number of surface atoms in the pure Pd sample foil

TPD peak area pure Pd foil = 2.076×10^{-9} a.u.

TPD peak area Pd/Zr catalyst = 2.586×10^{-9} a.u.

$$Number\ of\ active\ sites = \frac{1.2\times10^{16} \times 2.586\times10^{-9}}{2.076\times10^{-9}} \sim 1.49 \times 10^{16}\ \text{cm}^{-2}$$

$$n_{initial} = \frac{P_{initial} .V_{reactor}}{\text{RT}}$$

$$n_{initial} = \frac{5000\ \text{Pa} \times 2.96 \times 10^{-4} m^3}{8.314\ \text{J.K}^{-1}\ \text{mol}^{-1} \times 298\ \text{K}} \sim 5.97 \times 10^{-4}\ mol\ CO_2$$

$$n_{Consumed} = n_{initial} . X_{CO_2}$$

$$n_{Consumed} = 5.97 \times 10^{-4} \times 1 = 5.97 \times 10^{-4}\ mol$$

$$moles\ of\ CO_2\ \text{per sec} = \frac{n_{Consumed}}{\text{t}}$$

$$moles\ of\ CO_2\ \text{per sec} = \frac{5.97 \times 10^{-4}\ mol}{3000\ \text{sec}} = 1.99 \times 10^{-7}\ mol/s$$

$$Conversion\ of\ moles\ to\ molecules = 1.99 \times 10^{-7} \times 6.022 \times 10^{23} \sim 1.2 \times 10^{17}\ \text{sec}^{-1}$$

$$TOF = \frac{1.2 \times 10^{17}}{1.49 \times 10^{16}} \sim 8\ s^{-1} site^{-1}$$

$$molecules\ of\ CO_2\ consumed = 5.97 \times 10^{-4} \times 6.022 \times 10^{23} \sim 3.6 \times 10^{20}$$

$$TON = \frac{3.6 \times 10^{20}}{1.49 \times 10^{16}} \sim 2.4 \times 10^{4}\ site^{-1}$$

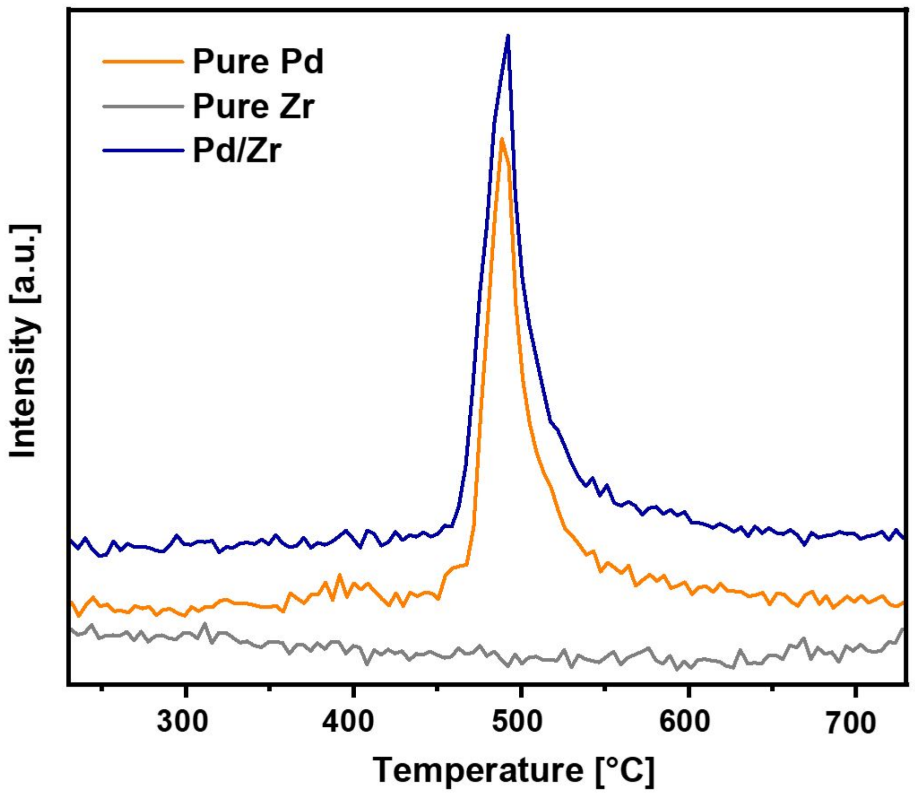

Figure S1. CO desorption profiles on the Pd/Zr catalyst after O_2 regeneration, on the pure Pd Foil, and the pure ZrO_2 foil.

The TPD data were used to quantify the number of active sites, which subsequently served as the basis for the turnover frequency (TOF) and turnover number (TON) calculations, detailed in Equation S1.

Table 1. Time evolution of turnover number (TON) and its first derivative during the reaction.

Time (s)	*TON*	*1st derivative of TON (s^{-1})*
**300**	2.4×10^3	10.6
**480**	4.8×10^3	16.6
**720**	9.6×10^3	20
**840**	1.2×10^4	15.5
**1020**	1.4×10^4	13.8
**1320**	1.9×10^4	10.2
**1860**	2.1×10^4	3.1
**300**	2.4×10^4	2.6

The first derivative of TON, representing the instantaneous turnover frequency (TOF), reaches a maximum value of 20 s^{-1} at 720 s, indicating the highest catalytic activity during the reaction cycle.

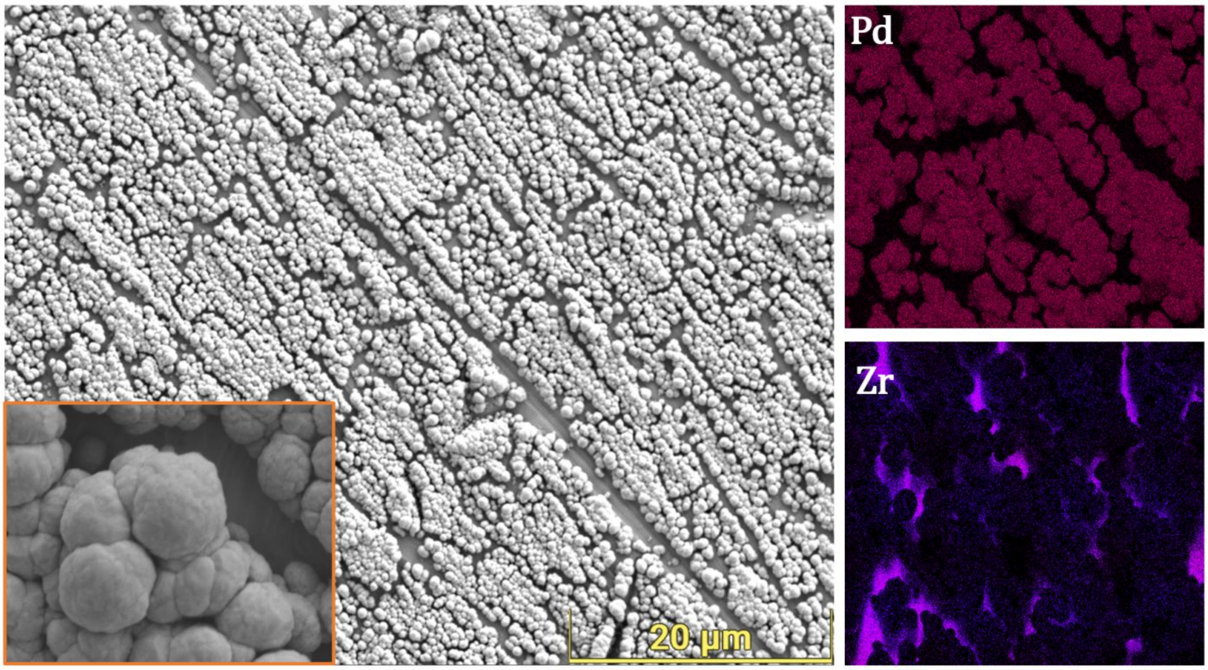

Figure S2. SEM image and EDX elemental maps of the as-deposited Pd/Zr surface before annealing.

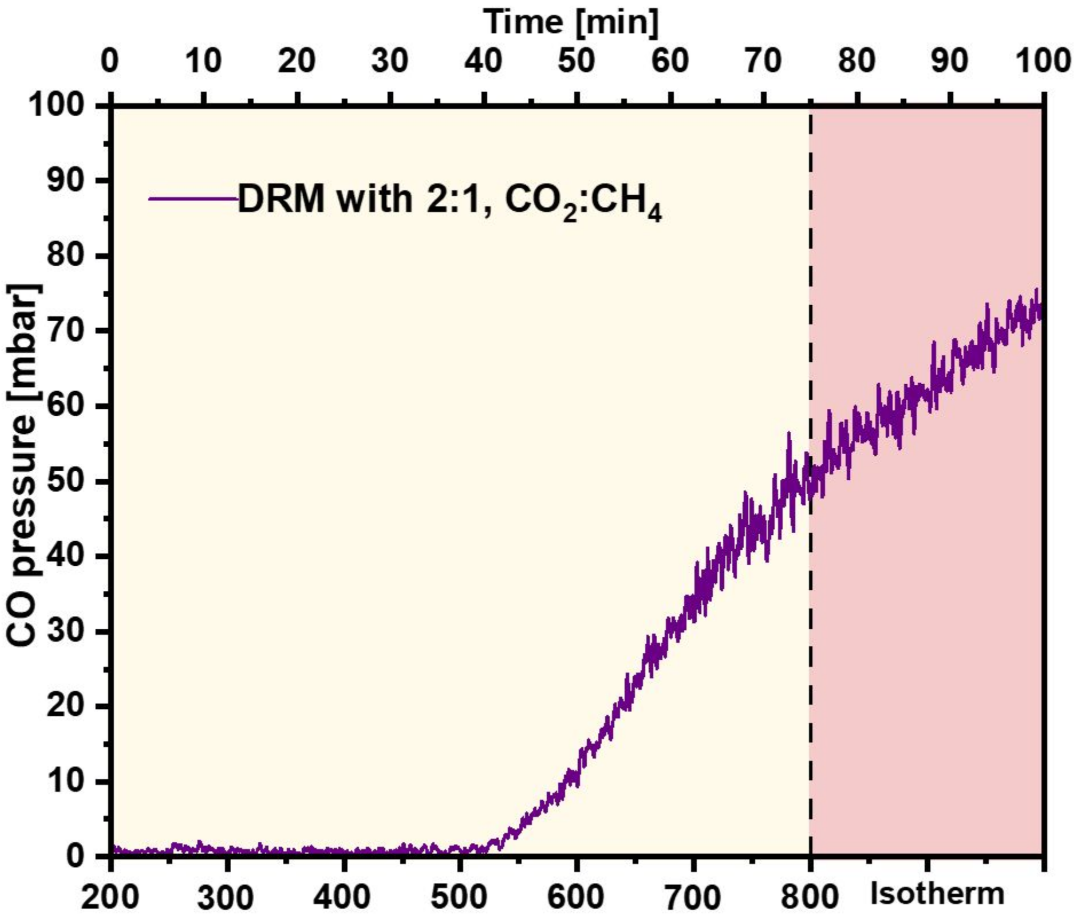

Figure S3. CO mass spectrometer signal during CO_2-rich DRM (CO_2:CH_4 = 2:1).

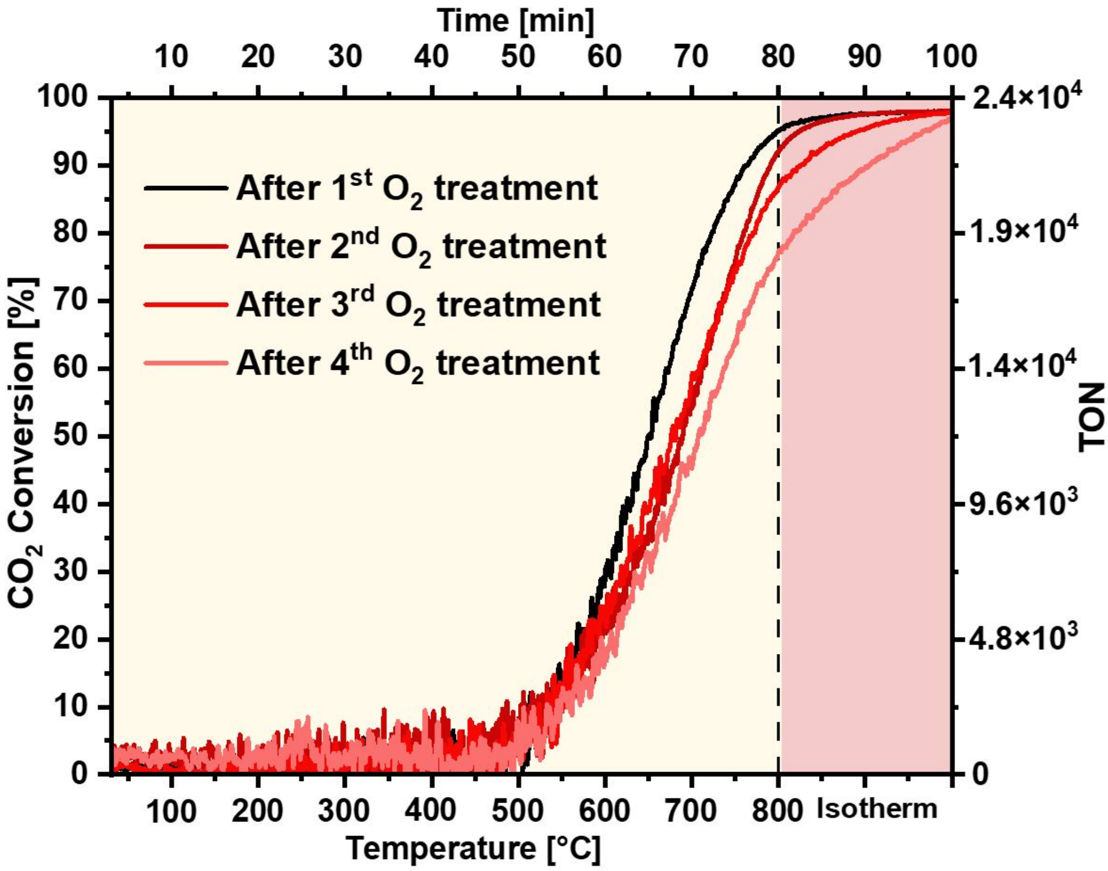

Figure S4. CO_2 conversion profiles for four consecutive DRM cycles, each performed after an O_2-based regeneration step.

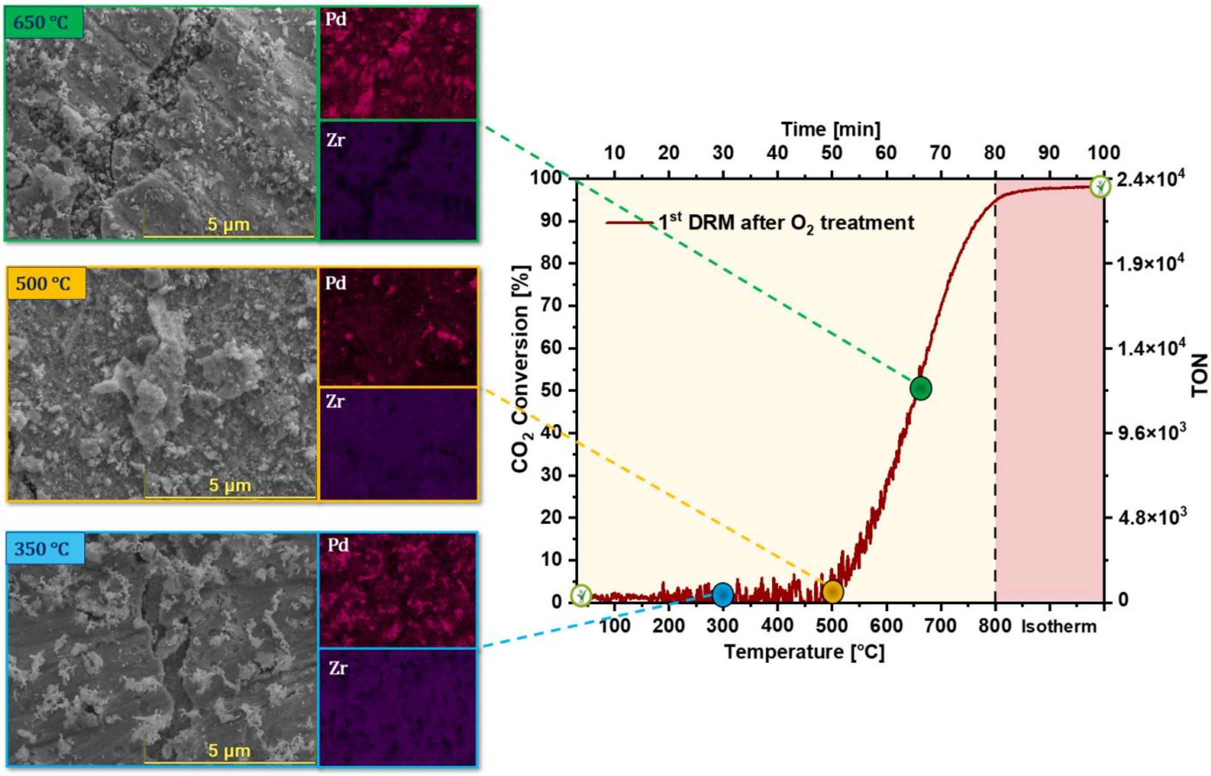

Figure S5. CO_2 conversion profile during the first DRM cycle post O_2 regeneration with corresponding SEM-EDX snapshots at 350 °C, 500 °C, and 650 °C. Reaction conditions: 50 mbar CH_4, 50 mbar CO_2, 977 mbar He, linear temperature ramp (10 °C min-1) up to 800°C, followed by isothermal reaction for 30 min.

To gain a deeper understanding of the structural evolution of the Pd/Zr catalyst during the first DRM cycle after O_2 regeneration, the reaction was halted at three key temperatures (350 °C, 500 °C, and 650 °C), and the surface was analyzed by SEM-EDX (**Figure S5**). At 350 °C, the surface remains largely free of carbon deposits, with Pd and Zr signals still clearly visible, indicating a clean and active interface. Remarkably, at 500°C-650 °C, previously identified as the "coking window" for the Pre-regeneration catalyst, the regenerated sample shows a markedly delayed onset of carbon formation and a more homogeneous Pd/Zr distribution. This progression highlights the stabilizing effect of O_2 treatment, also indicating

that the regenerated Pd/Zr interface resists coking initiation more effectively, at least within the early stages of the DRM reaction.
